# Ccdc3: A New P63 Target Involved in Regulation Of Liver Lipid Metabolism

**DOI:** 10.1038/s41598-017-09228-8

**Published:** 2017-08-21

**Authors:** Wenjuan Liao, Hongbing Liu, Yiwei Zhang, Ji Hoon Jung, Jiaxiang Chen, Xiaohua Su, Yeong C. Kim, Elsa R Flores, San Ming Wang, Malwina Czarny-Ratajczak, Wen Li, Shelya X. Zeng, Hua Lu

**Affiliations:** 1 Department of Biochemistry & Molecular Biology and Tulane Cancer Center, Tulane University School of Medicine, New Orleans, LA 70112 USA; 2 Department of Molecular Oncology, Cancer Biology and Evolution Program, H. Lee Moffitt Cancer Center, Tampa, Florida USA; 3 Department of Genetics, Cell Biology and Anatomy, University of Nebraska Medical Center, 986805 Nebraska Medical Center, Omaha, 68198 NE USA; 4 Department of Medicine, Center for Aging, Tulane University School of Medicine, New Orleans, LA 70112 USA; 5 Laboratory of General Surgery, The First Affiliated Hospital, Sun Yatsen University, Guangzhou, Guangdong 510080 P.R. China; 6 Present Address: Department of Pediatrics, Tulane University School of Medicine, New Orleans, LA 70112 USA; 7 Department of Physiology, Present Address: Jiangxi Medical College of Nanchang University, Nanchang, Jiangxi 330006 P.R. China; 8 Faculty of Health Sciences, University of Macau, Macau, China

## Abstract

TAp63, a member of the p53 family, has been shown to regulate energy metabolism. Here, we report coiled coil domain-containing 3 (CCDC3) as a new TAp63 target. TAp63, but not ΔNp63, p53 or p73, upregulates CCDC3 expression by directly binding to its enhancer region. The CCDC3 expression is markedly reduced in TAp63-null mouse embryonic fibroblasts and brown adipose tissues and by tumor necrosis factor alpha that reduces p63 transcriptional activity, but induced by metformin, an anti-diabetic drug that activates p63. Also, the expression of CCDC3 is positively correlated with TAp63 levels, but conversely with ΔNp63 levels, during adipocyte differentiation. Interestingly, CCDC3, as a secreted protein, targets liver cancer cells and increases long chain polyunsaturated fatty acids, but decreases ceramide in the cells. CCDC3 alleviates glucose intolerance, insulin resistance and steatosis formation in transgenic CCDC3 mice on high-fat diet (HFD) by reducing the expression of hepatic PPARγ and its target gene CIDEA as well as other genes involved in de novo lipogenesis. Similar results are reproduced by hepatic expression of ectopic CCDC3 in mice on HFD. Altogether, these results demonstrate that CCDC3 modulates liver lipid metabolism by inhibiting liver de novo lipogenesis as a downstream player of the p63 network.

## Introduction

Liver, as a primary metabolic organ, plays a vital role in the regulation of lipid metabolism and is sensitive to energy intake and vulnerable to metabolic disorder-causing stressors or conditions. Nowadays, the most common cause of liver dysfunctions in the United States and other western industrialized countries is nonalcoholic fatty liver disease (NAFLD), representing over 75% of the chronic liver disease^1^. NAFLD exhibits a broad spectrum of conditions ranging from simple steatosis to nonalcoholic steatohepatitis (NASH), fibrosis and cirrhosis, which may ultimately progress to hepatocellular carcinoma. The steatosis rate reveals the imbalance between input (lipolysis in white adipose tissue and de novo lipogenesis), and output (β-oxidation and secretion) of free fatty acids in hepatic tissues^2^. Insulin resistance plays a major role in the development of NAFLD, while ectopic liver lipid exacerbates hepatic insulin resistance, promotes systemic inflammation, and increases the risk of developing both type 2 diabetes mellitus and cardiovascular disease^3, 4^. Although extensive research has been conducted in this area, the complexly interlocked molecular events and related cellular behaviors that occur during the initiation and progression of hepatic steatosis are not entirely understood. A recent study revealed p63 as a key regulator in liver metabolism^5^.

p63 is the most ancient member of the p53 family involved in multiple facets of biology, including embryonic epidermal development, cell proliferation, differentiation, survival, apoptosis, senescence, and aging^6, 7^. Because of the presence of two promoters, p63 encodes two major classes of proteins: those containing a transactivating (TA) domain homologous to the one present in p53 (TAp63) and those that lack the TA domain (∆Np63). Also, the C-terminal alternate splicing generates at least three p63 variants (α, β and γ) in each class^8^. Among these isoforms, TAp63 was shown to control various aspects of metabolism^5^. TAp63 knockout (TAp63KO) mice more rapidly developed liver steatosis and insulin intolerance than did wild-type mice. Also, TAp63KO mouse embryonic fibroblasts (MEFs) showed defective glucose uptake. Although several key metabolism regulators were identified as TAp63 direct targets, such as Sirt1, AMPK, and LKB1^5^, the precise molecular mechanisms underlying hepatic steatosis remain largely elusive.

In the present study, we identified the CCDC3-encoding gene as a novel target for TAp63, which is involved in lipid metabolism. CCDC3 (we will use CCDC3 for its protein form here) is a recently discovered secretory protein that is mainly expressed in endothelial cells and adipose tissues and highly conserved among different species^9, 10^. CCDC3 mRNA expression in adipocytes and endothelial cells is regulated by hormones and nutritional factors^9^. A study showed that CCDC3 could repress TNF-α/NF-KB-induced a pro-inflammatory response in endothelial cells, suggesting a potential role for CCDC3 in the development of obesity and atherosclerosis^11^. As detailed below, our study using cellular analyses and two mouse model systems with ectopic CCDC3 expression unveils CCDC3 as an authentic transcriptional target of TAp63 to play a role in the regulation of liver lipid metabolism.

## Results

### Identification of CCDC3 as a novel target specific for TAp63

**Figure 1:**
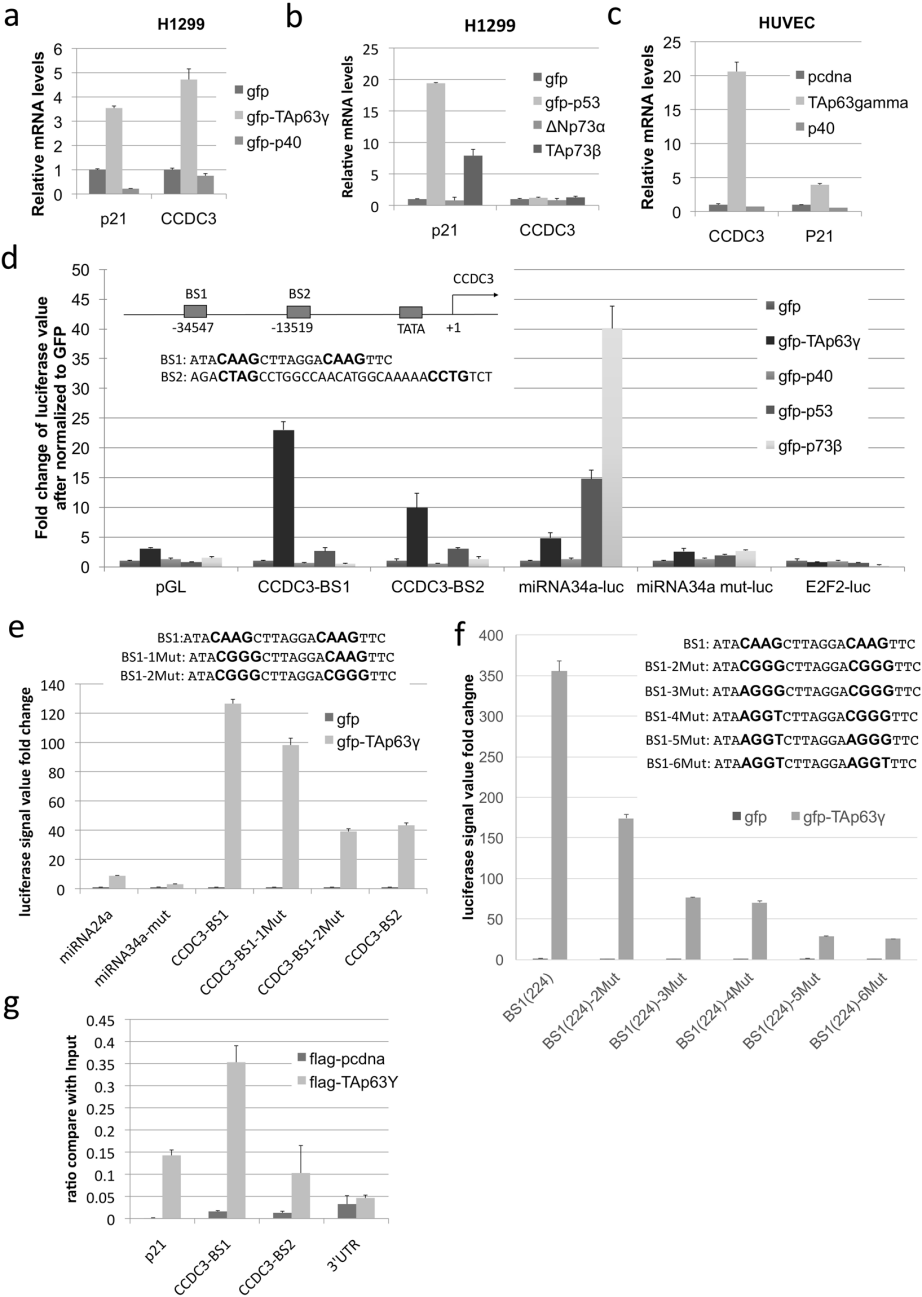
TAp63, but not ΔNp63, p53, p73, transcriptionally activates CCDC3. (**a–c**) qRT-PCR showing that overexpression of TAp63gamma, but not ΔNp63, p53 or p73, could induce CCDC3 in H1299 (**a**,**b**) and HUVEC cells (**c**). (**d,f**) Schematic representation of two p63 binding sites (BS1 and BS2) and mutated binding sites (BS1-1Mut, BS1-2Mut, BS1-3Mut, BS1-4Mut, BS1-5Mut, BS1-6Mut) at the upstream of the transcriptional initiation site of the CCDC3 gene. H1299 cells were co-transfected with the indicated plasmids and β-Gal plasmid. Forty eight hours after transfections, cells were harvested for luciferase assays. Luciferase activity was normalized against β-Gal expression. Data are presented as means ± S.D., n = 3. (**g**) ChIP assays after HaCat cells were treated with metformin using the indicated antibodies followed by q-PCR for CCDC3 promoter sequences or negative control sequences (3′-UTR). Data are presented as means ± S.D., n = 3.

Inauhzin (INZ) is a small molecule identified in our lab to induce p53 as well as p73^12^. In searching for new INZ-responsive transcriptional targets of these p53 family members, we identify CCDC3 as a potential target of TAp63 through microarray analysis of RNAs extracted from cells treated with or without INZ. Initially, CCDC3 mRNA level was found to be induced upon INZ treatment of p53^+/+^ HCT116 colon cancer cells (Supplementary Fig. 1a), suggesting that CCDC3 might be a potential p53 target. However, surprisingly, overexpression of neither p53 nor p73 could induce CCDC3 in this cell line (data not shown). By contrast, ectopic expression of TAp63γ, a p63 isoform that has the strongest transcriptional activity among all the splice variants, dramatically induced the expression of CCDC3 and p21 (as a control) at RNA levels in p53-null H1299 lung adenocarcinoma cells, but the induction was not observed when the cells overexpressed p40 (Fig. 1a), which is a shorter and transcriptionally inactive isoform of p63 without the TA domain^13, 14^. To further exam if the induction of CCDC3 is p63 dependent or not, we overexpressed different p63 isoforms as well as p53 or p73 in H1299 cells and analyzed CCDC3 mRNA levels. Again, only ectopic TAp63γ, but not p53 or p73, increased CCDC3 expression (Fig. 1b). Similar results for ectopic TAp63γ and p40 were also observed in p53-null HCT116 and SaoS2 cancer cells (Supplementary Fig. 1b and c). Because CCDC3 was previously shown to be highly expressed in mouse endothelial cells^9, 11^, we also tested if TAp63 could induce CCDC3 expression in normal endothelial cells using human umbilical vein endothelial cells (HUVEC). Consistently with the results using cancer cells above, TAp63γ, but not p40, also induced the expression of CCDC3 mRNA in HUVEC (Fig. 1c). These results demonstrate that CCDC3 can be specifically induced by TAp63γ, but not p73, p53 or Delta-N forms of p63, in both normal and cancer cells, and indicate that CCDC3 might be a specific transcriptional target of TAp63γ.

Analysis of the human CCDC3 promoter and enhancer region revealed two p63 consensus DNA binding elements located at 34547 (BS1) and 13591 (BS2) nucleotides upstream from the transcription start site (Fig. 1d). To test if TAp63γ could bind to any of these sites, we performed luciferase reporter assays. Indeed, TAp63γ activated the CCDC3-luciferase reporters in both BS1 and BS2 with an apparent preference to BS1, but p53, p73 and p40 were unable to activate the reporter luciferase (Fig. 1d), when their expression levels were equivalent (Supplementary Fig. 1d). We also found a mutation of this site from p53/p63 binding active sequences to repressive sequences markedly reduced the luciferase activity (Fig. 1e). The BS1–2Mut mutation still keep around 30% of luciferase reporter gene expression compare to original BS1 construct. We speculated that TAp63 might also bind to other sequences on this 1098 bp enhancer region except the p53/p63 conserved binding sequences. To test this possibility, we generated another plasmid construction BS1(224), which only contains 224 bp of CCDC3 enhancer region. Our luciferase reporter assay showed that the BS1(224) displays a stronger luciferase activity than does the original BS1 (Supplementary Fig. 1e). We also confirmed that activation of the reporter luciferase on BS1(224) is specific to TAp63, but not p53 and p73 (Supplementary Fig. 1f). Although our BS1 (224)-2 Mut mutant still retained ~30% luciferase activity, further mutagenesis demonstrated that the mutant with the mutation of all the conserved 8 nucleotides(BS1-6Mut) on the canonical p53/p63 binding sequences could markedly reduce the luciferase activity by more than 90% (Fig. 1f, the BS1 (224)-6Mut). These results demonstrate that the binding of TAp63γ to the BS1 site is specific and also suggest that the CCDC3 promoter region harbors a unique TAp63-binding element specific to TAp63, but not p53 and p73.

The binding of TAp63γ to the putative responsive elements within the CCDC3 enhancer was also confirmed by chromatin immunoprecipitation (ChIP) analysis when HaCat cells, an immortal keratinocyte cell line^15^, with overexpression of flag-TAp63γ were treated with metformin to activate TAp63 transcriptional activity^5^ (Fig. 1f). Consistently, TAp63γ preferred to binding to the BS1 site though it bound to both of the BS1 and BS2 sites within the CCDC3 enhancer in comparison with the nonspecific site (Fig. 1f). These results indicate that TAp63 prefers targeting the BS1 element of the CCDC3 promoter.

**Figure 2:**
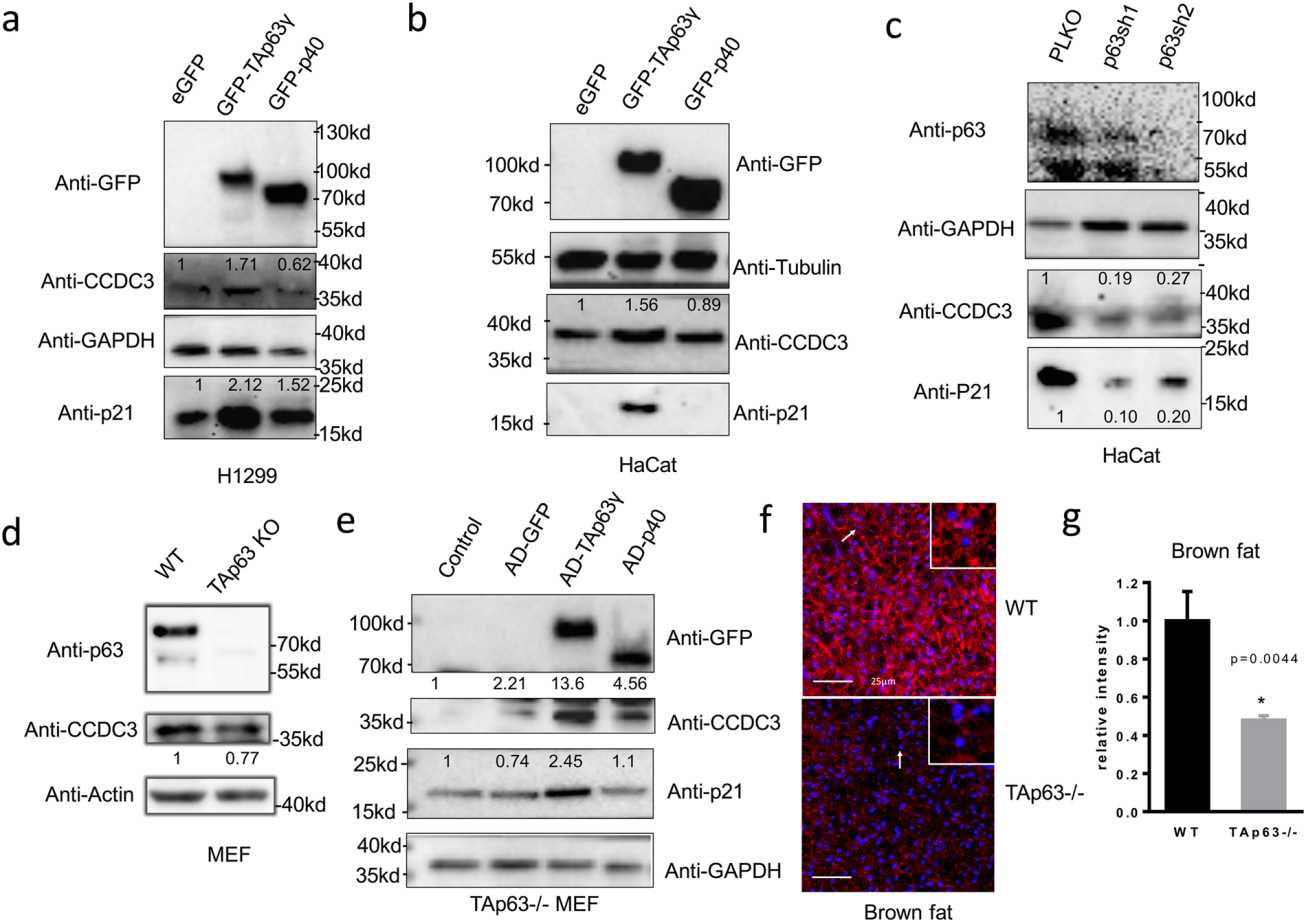
CCDC3 protein level is induced by overexpression of TAp63 and decreased by knockdown or knockout of TAp63. (**a,b**) WB analysis showing that overexpression of TAp63 in H1299 cells (**a**) and HACAT cells (**b**) can induce CCDC3 protein level, while p40 inhibits CCDC3 expression. (**c**) p63 knockdown decrease CCDC3 protein level in HaCat cells. (**d**) WB analysis showing CCDC3 protein level is lower in TAp63^−/−^ MEF cells than that in WT MEF cells. (**e**) TAp63^−/−^ MEF cells infected with GFP (control), TAp63, or p40 adenovirus as indicated followed by WB analysis with indicated antibodies. (**f**) IF staining for CCDC3 (red) and DAPI (blue) using the brown adipose tissue from the indicated WT and TAp63^−/−^ mice. (**g**) Quantification of the intensity of CCDC3 staining from Fig. 2f. Densitometry analysis is labeled on top of each band.

Since the above studies only showed the TAp63γ-induced RNA level of CCDC3 (Fig. 1; Supplementary Fig. 1), we checked if this induction would lead to the increase of CCDC3 protein level as well by western blot (WB) analysis. Indeed, CCDC3 protein level was increased by ectopic TAp63γ, but not p40, in H1299 (Fig. 2a) and HaCat cells (Fig. 2b). Conversely, knockdown of endogenous TAp63 by shRNA led to the marked decreased of endogenous CCDC3 in HaCat cells (Fig. 2c). Consistent with these results, TAp63KO MEF cells also showed a marked reduction of CCDC3 protein levels (Fig. 2d), which was induced by ectopic TAp63γ, but not p40, in the cells (Fig. 2e) (of note, CCDC3 was also slightly increased by adenovirus-GFP, which could be a non-specific effect by adenovirus infection). Interestingly, CCDC3 was drastically decreased in brown fat tissues of TAp63KO mice compared with that in wild-type mice (Fig. 2f,g). Taken together, these results (Figs 1 and 2) demonstrate that CCDC3 is a bona fide transcriptional target of TAp63γ, but not other members of the p53 family, as further validated in the following experiments.

### TAp63 regulates CCDC3 in response to different stimuli

**Figure 3:**
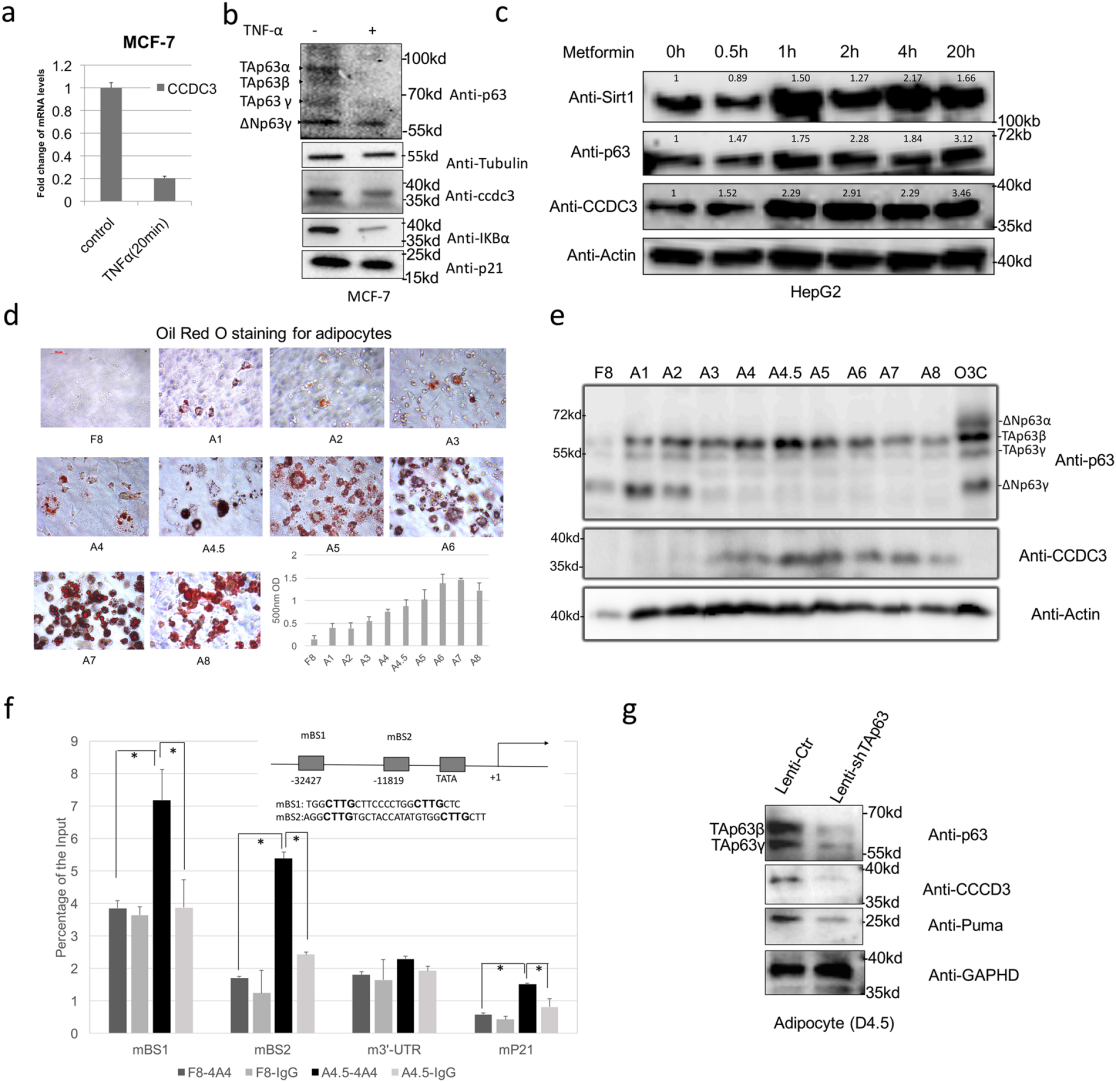
The expression of CCDC3 is well correlated with TAp63γ levels upon different drug treatment and during adipocyte differentiation. (**a**,**b**) MCF-7 cells treated with TNFα or DMSO at 20 ng/ml for 20 min and harvested for qRT-PCR (**a**) and WB (**b**) analyses to detect CCDC3 level. (**c**) HepG2 cells treated with 1 mM metformin at the time points as indicated and harvested for WB analysis to determine protein levels with indicated antibodies. Densitometry analysis is labeled on top of each band. (**d**) 3T3-L1 preadipocytes undergo differentiation to adipocytes. After incubation in differentiation media for 8 days, 3T3-L1 adipocytes were stained with Oil Red O. The absorbance of the extracted Oil Red O was spectrophotometrically determined at 570 nm to measure triglyceride (TG) accumulation. The error bars represent the standard error of the mean. (**e**) Cells after incubation in differentiation media for different days as shown in panel d were harvested for WB analysis with indicated antibodies. (**f**) ChIP assays of undifferentiated (F8) and differentiated (A4.5) adipocytes using the indicated antibodies followed by q-PCR for CCDC3 promoter sequences or negative control sequences (3′-UTR). Data are presented as means ± S.D., n = 3. (**g**) 3T3-L1cells infected with shTAp63 lentivirus and control virus at 2.5 days after differentiation. At 2 days after infection (Day 4.5 after differentiation), cells harvested for WB analysis to determine protein levels with indicated antibodies.

Next, we determined if CCDC3 expression is responsive to reagents or signals that affect TAp63 activity. Since our previous study showed that TNFα inhibits TAp63 transcriptional activity^15^, we first tested if TNFα could also inhibit CCDC3 expression. Indeed, this was the case, as treatment of MCF7 cells with TNFα led to the suppression of CCDC3 expression at both the mRNA and protein levels (Fig. 3a and b). The reduction of CCDC3 expression was due to the knockdown of TAp63s, but not the Delta-N forms of p63, as the latter failed to activate the expression of CCDC3 (Figs 1–2). Inversely, Metformin, a clinical drug used for type 2 diabetes and previously shown to stimulate TAp63 expression^5^, induced the protein level of CCDC3, which was well correlated with the induction of TAp63 and one of its targets, Sirt1, in a time-dependent manner (Fig. 3c). Because TAp63 has been linked to lipid metabolism^5^, and CCDC3 is highly expressed in adipose tissues^9^, we also assessed the protein level of TAp63 and CCDC3 during adipocyte differentiation by employing NIH3T3-L1 preadipocytes. As shown in Fig. 3d, the NIH3T3-L1 cells underwent adipocyte differentiation, starting from day one after incubation in differentiation media. Remarkably, both of the CCDC3 and TAp63 protein levels were concomitantly unregulated during the mouse adipocyte differentiation (Fig. 3e). Conversely, ∆Np63 was downregulated during adipocyte differentiation (Fig. 3e). Interestingly, the expression of CCDC3 started on day 3 (A3) when the level of ΔNp63γ drastically decreased, suggesting that ΔNp63γ might play a dominant negative role in suppression of TAp63 transcriptional activity as previously shown in other cell systems^16^. Because TAp63β appeared to be the major isoform of p63 during the adipocyte differentiation (Fig. 3e), we also tested if TAp63β could increase CCDC3 level. Indeed, similar to TAp63γ, ectopic expression of TAp63β increased the expression of CCDC3 at both mRNA and protein levels in H1299 cells (Supplementary Fig. 2a and b). Based on these results, CCDC3 is specifically regulated by TAp63 but not ∆Np63 in adipocye. Therefore, we performed a ChIP analysis using an anti-p63 antibody in both undifferentiated (F8) and differentiated (A4.5) adipocytes. Consistent with the ChIP result using ectopic TAp63 in HaCat cells (Fig. 1g), our ChIP results in adipocytes further verified the binding of endogenous TAp63 to both of the BS1 and BS2 sites with preference to the BS1 within the CCDC3 enhancer in differentiated (A4.5), but not undifferentiated (F8), adipocytes (Fig. 3f). In line with this ChIP result, knockdown of endogenous TAp63s with a short hairpin RNA (shRNA) for TAp63 at Day 4.5(A4.5) differentiated adipocytes also led to the marked reduction of endogenous CCDC3 protein levels in these cells (Fig. 3g and Supplementary Fig. 2c). A semiquantitative RT-PCR was conducted to confirm the knockdown of TAp63 in mRNA level (Supplementary Fig. 2d). Together, these results demonstrate that CCDC3 expression is responsive to TAp63 activation or inactivation upon treatment with different agents or signals, and once again verify CCDC3 as a physiological target gene of TAp63.

### CCDC3 modifies lipid metabolism in liver cancer cells

**Figure 4:**
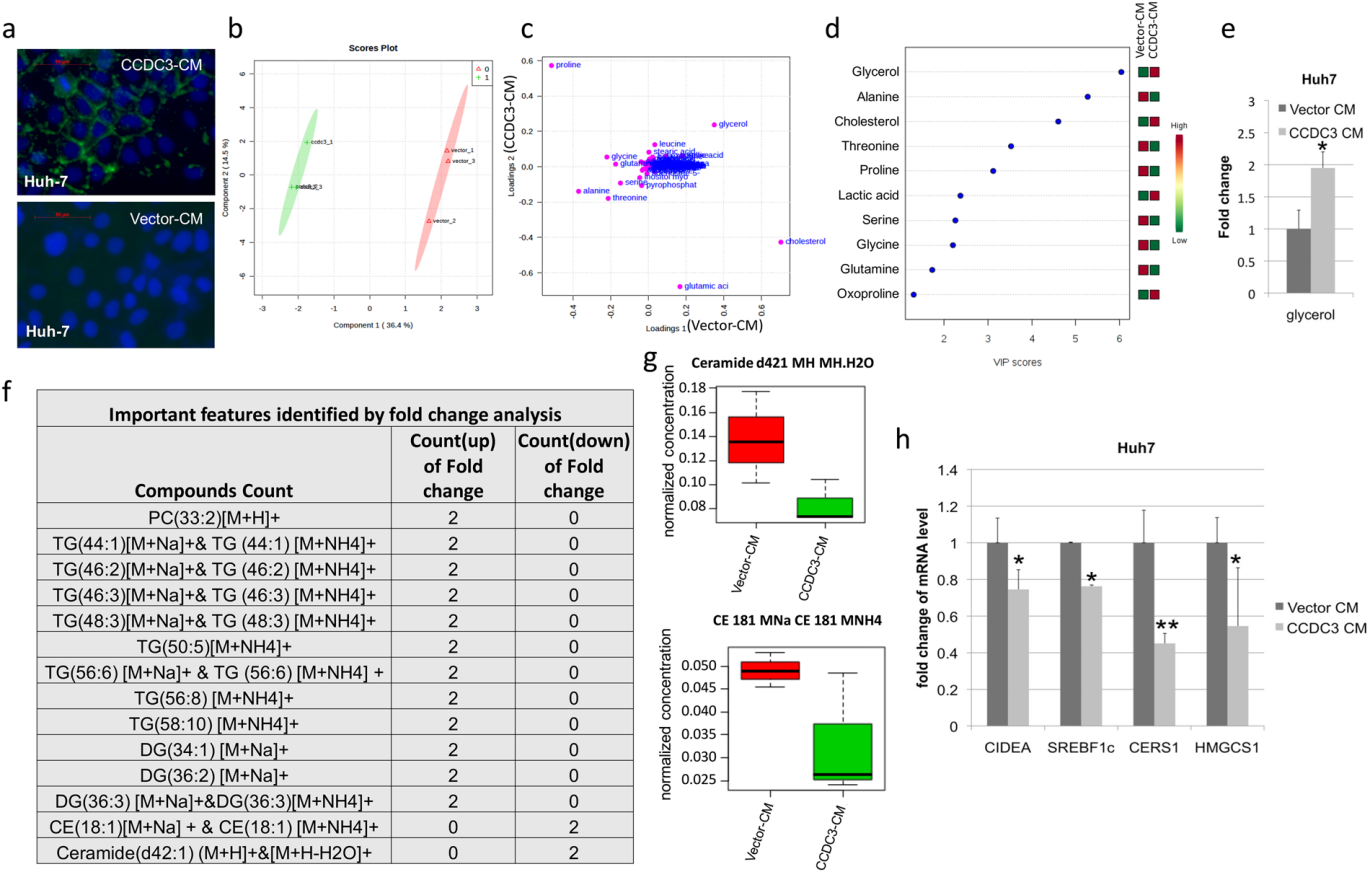
CCDC3 recognizes liver cells, and increases long chain polyunsaturated fatty acids (PUFC) level and decreases ceramide level. (**a**) Representative images of anti-myc immunofluorescence of Huh-7 cells cultured with CCDC3 conditioned medium or control medium. Monoclonal antibody against Myc (green) merged with DAPI (blue). (**b**) Partial least squares-discriminant analysis (PLS-DA) score plots of component 2 versus component 1 for CCDC3-CM area (green) and vector-CM area (red) treaded Huh7 samples. (**c**) PLS-DA loading plot comparing variables between vector-CM (loadings 1) and CCDC3-CM (loadings 2) treated Huh7 samples. The variables (metabolites) in cell samples with Variable Importance in Projection (VIP) values of more than 1.0 are marked in pink. (**d**) Top 10 metabolites modified after CCDC3-CM treated through PLS-DA analysis based on the VIP values > 1.0. (**e**) glycerol levels in two groups presented in a fold change manner. Results are shown in means ± SE. *P < 0.05. (**f**) Important lipid metabolites identified by fold change analysis. The values are plotted in log2 scale. List of abbreviations in the table: CE: Cholesteryl ester (CE), PC: Phosphtatidylcholines, TG: Glyceryl tristearate, DG: Distearoylglycerol. (**g**) Ceramide and CE 18:1 concentration change in between CCDC3-CM (1) group and Vector-CM (0) group. (**h**) mRNA expression of lipogenic and fatty acid oxidation genes in the Huh7 cells treated with CCDC3 or control media. *P < 0.05, **P < 0.01.

Given the fact that CCDC3 can be secreted out of cells^9^, as also confirmed by our lab (Supplementary Fig. 3a–c), we speculated that CCDC3 might have an endocrine activity by targeting distant tissues or cells. To identify its target cells, we screened various cell lines available in the lab by examining if CCDC3 could bind to the surface of the cells using immunofluorescence (IF) staining. To do so, 293-HEK stable cells expressing Myc-CCDC3 (OE CCDC3) or control vector were generated (Supplementary Fig. 3a–c). We harvested media from the cultured 293-HEK stable cells with OE Myc-CCDC3 and control vector, respectively, and then treated various cell lines with either of the Myc-CCDC3-containing and Myc-CCDC3-free media (Supplementary Fig. 3d) followed by IF staining with the anti-Myc antibody. Among all the cells incubated in the Myc-CCDC3 media (Supplementary Fig. 3e and f), liver cancer cells, such as Huh-7 (Fig. 4a), HepG2 and PLC-PRF-5 cells (Supplementary Fig. 3e), showed specific IF signals lined up on their cytoplasmic membrane. Other cells with positive IF signals included breast cancer MCF7 and MDA-MB-231 cells, osteosarcoma U2OS and SaoS2 cells, and normal human fibroblast NHF cells (Supplementary Fig. 3e). However, a number of cells showed negative IF signals, including HeLa, MEF, 293HEK, pre-brown adipocyte, SF767, H1299, HCT116, Panc-1, Mia-paca2 and WI-38 cells (Supplementary Fig. 3f). These results suggest that CCDC3 might selectively target particular types of mammalian cells.

Since liver cancer cells were clearly positive with Myc-CCDC3 staining on their cytoplasmic membrane (Fig. 4a), and liver plays a major role in metabolism^17^, we suspected that normal liver cell might be one of the main target cells for CCDC3. Thus, we decided to first determine if CCDC3 could affect lipid metabolism in hepatic cancer cells by performing metabolomics analysis. GC-QTOF MS analysis was conducted on cell samples treated with CCDC3 medium and control medium. Then, mutltivariate analysis was performed for 135 metabolites based on the mass spectrometry data. The partial-least-squares discrimination analysis (PLS-DA) score plots^18^ showed two cluster between CCDC3-treated (green area) and the control sample (pink area) (Fig. 4b). This result suggested changes in metabolites of CCDC3 treated samples. The significance of the metabolites change was ranked using the variable importance in projection (VIP) score (>1)^18^ from the PLS-DA model that was responsible for the separation of CCDC3 conditioned medium treated samples from control samples. From this analysis, we identified ~20 compounds with VIP values of more than 1.0 (Fig. 4c). Top 10 candidates as listed in Fig. 4d and Supplementary Fig. 4a included glycerol, alanine, cholesterol, threoinine, proline, lactic acid, serine, glycine, glutamine and xoproline. Interestingly, glycerol level was significantly increased in CCDC3-treated Huh-7 cells, compared to the cells treated with CCDC3-free media (Fig. 4e). Glycerol is an important metabolite of lipid metabolism. To further understand the lipid changes upon CCDC3 treatment, we further did a lipidomics study and analyzed all the lipids using LC-MS. We conducted multivariate statistical analysis of 88 lipids (or variables). Through analysis of the fold change (count with fold change >2 or <2, Supplementary Fig. 4b) and PLS-DA analysis (VIP value more than 1, Supplementary Fig. 4c), we identified top 14 lipid compounds with significant change upon CCDC3 treatment and also found a decrease of ceramide level and an increase of the level of long chain polyunsaturated fatty acids (PUFAs) (Fig. 4f and g). The reduction of ceramide and the elevation of PUFAs have been shown to be associated with fatty liver diseases^19–24^. To explore the potential mechanism underlying the regulation of lipid metabolism by CCDC3, we detected the expression of several key genes involved in fatty acid uptake, lipid storage, lipolysis, and thermogenesis. Among all those genes, some of the genes involved in de novo lipogenesis were down-regulated when cells were incubated with Myc-CCDC3-containing media (Fig. 4h), including cell death activator-A (CIDEA), sterol regulatory element binding protein 1c (SREBF1c), ceramide synthase (CERS1), and 3-hydroxy-3-methylglutaryl-CoA synthase 1 (HMGCS1). These results suggest that CCDC3 may inhibit de novo lipogenesis by down-regulating the expression of the genes involved in this process.

### Generation of CCDC3 transgenic mice

**Figure 5:**
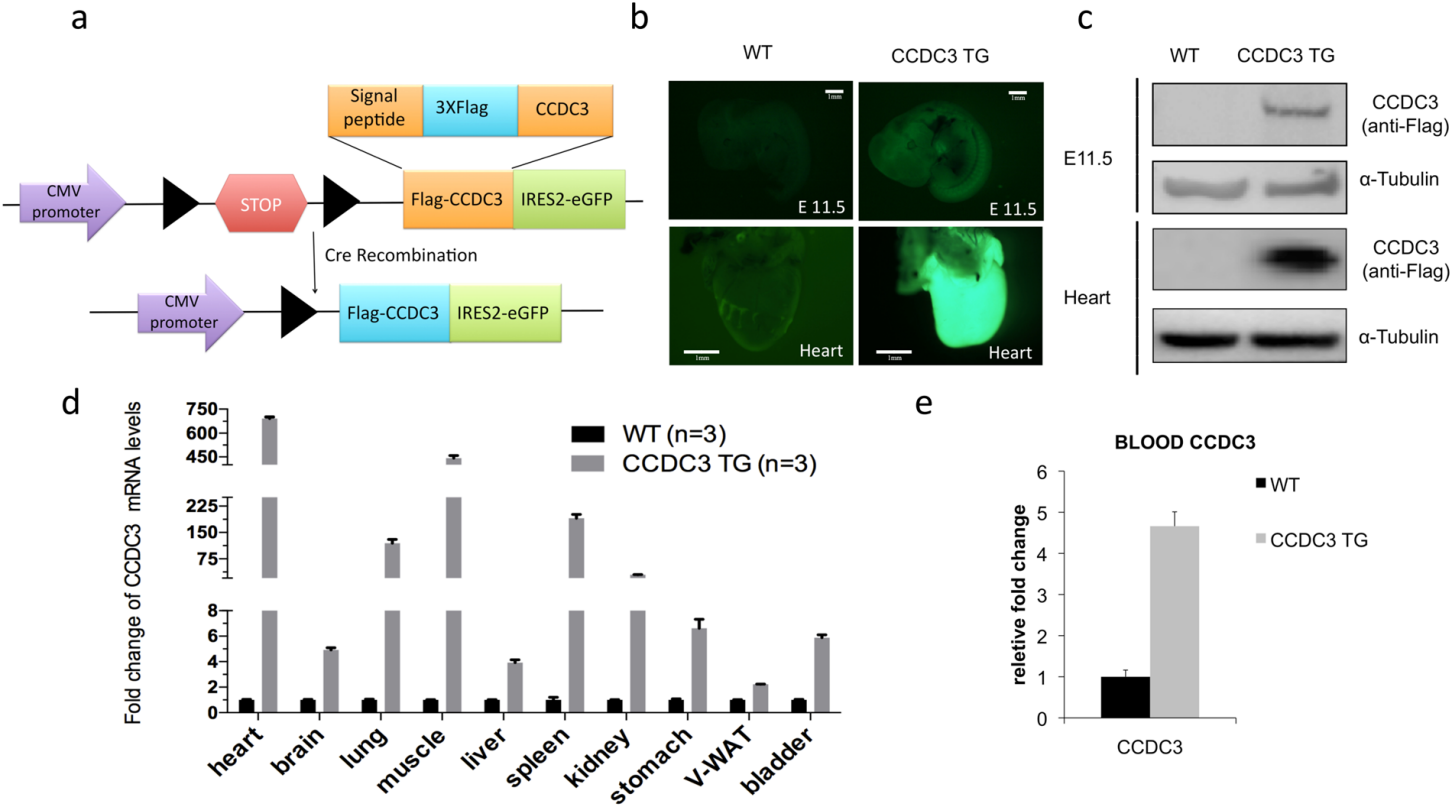
CCDC3 is ubiquitously overexpressed in transgenic mice after CMV-Cre mediated DNA recombination. (**a**) Schematic showing the construct of CCDC3 transgenic mice. Flag-mouse CCDC3 was placed downstream of a loxP-flanked Stop cassette and linked to a GFP which transcribed from IRES promoter. Following Cre-mediated excision of Stop cassette, Flag-ccdc3 is expressed. (**b**) Representative images of GFP expression in a whole E11.5 embryo and an isolated heart from a two days old mouse, taken under an anatomic microscope, confirming the global expression of CCDC3 in TG mice. (**c)** WB analysis confirms the CCDC3 protein express in E11.5 embryos and heart tissues of TG mice. (**d**) qRT-PCR verifies the expression of CCDC3 in different tissues of adult TG mice. (**e**) ELISA confirms the high level of CCDC3 in the blood serum of adult TG mice. Data represent the mean ± SD. *P < 0.05.

To better understand the biological function of CCDC3 in the regulation of liver lipid metabolism, we decided to generate a CCDC3 transgenic (TG) mouse model. Transgenic DNA fragments containing the chicken β-actin promoter/CMV enhancer, the loxP flanked STOP cassette and Flag-mouse CCDC3 coding sequence followed by the IRES2-EGFP sequence and a rabbit β-globin polyA tail were used for pronuclei injection. Using this strategy, we have generated three independent CCDC3-TG mouse founders. Flag-CCDC3 was universally overexpressed in transgenic mice after CMV-Cre-mediated excision of the Stop cassette (Fig. 5a). For all three overexpression lines, transgenic mice were born in Mendelian frequencies and found to be healthy and fertile. As presented in Fig. 5b–d and Supplementary Figs. 5a,b for one of the two mouse lines, the expression of both CCDC3 and GFP was detected in the entire embryo at day 11.5 and all of the organs or tissues through real-time QPCR and/or WB analyses. Flag-CCDC3 was also detected in the blood of adult mice by an ELISA assay (Fig. 5e). These results confirm that we have successfully generated Flag-CCDC3-TG mice.

### CCDC3 transgenic mice display decreased responsiveness to high fat diet

Next, we analyzed potential global effects of exogenous CCDC3 on the development, growth, and metabolism of mice. After 12 months on a standard chow diet, CCDC3 TG mice displayed no apparent changes in total body weight (Supplementary Fig. 6a), food intake, and energy expenditure, compared with control mice from the same breeding colony (data not shown). Also, their blood glucose and fasting serum insulin levels did not show any significant change compared to that in control mice. Interestingly, glucose intolerance was improved in CCDC3 TG mice (Supplementary Fig. 6b), although their insulin sensitivity was not altered in comparison with that of control mice (Supplementary Fig. 6c). These results indicate that overexpression of CCDC3 in mice on a standard chow diet does not have any significant impact on the development, growth, and metabolism of the animals, but could significantly improve their glucose tolerance.

**Figure 6:**
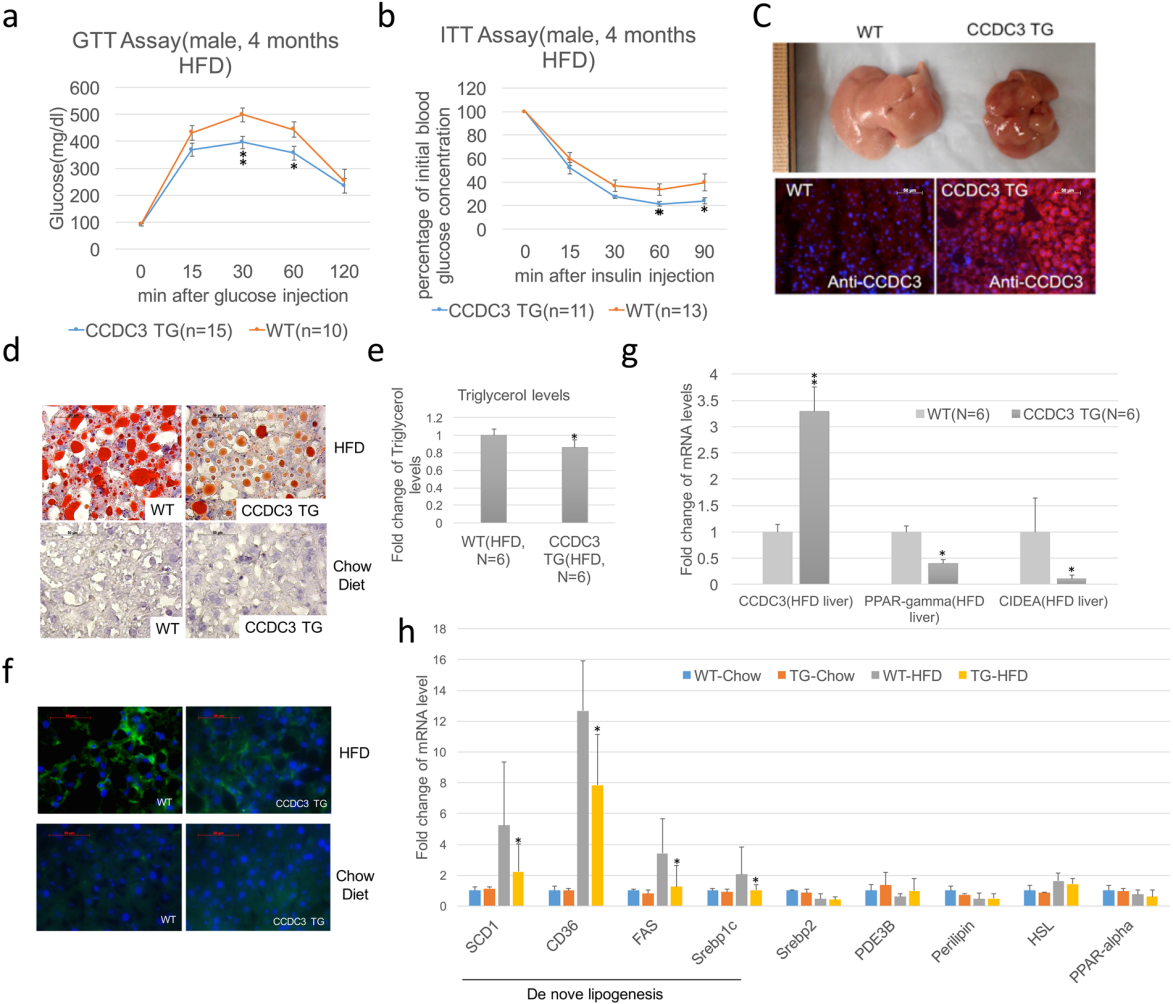
Overexpression of CCDC3 alleviates glucose intolerant, insulin resistant and fatty liver formation. (**a,b**) Glucose tolerance test (**a**) and Insulin intolerance test (**b**) were performed using 5-month-old WT and CCDC3 TG mice on high-fat Diet (HFD) for 4 months, each point on the graph indicates the level of glucose in the blood at the indicated time point on the x-axis. (**c**) Representative photographs of fatty liver from mice in groups on HFD (upper images), and CCDC3 immuoflurorescence staining images from the same liver sections (lower images, polyclonal antibodies again CCDC3(Red) merged with DAPI(blue). (**d**) Representative Images of mouse liver sections after oil red O staining, × 20 magnification. Red staining indicates lipid deposits. (**e**) Hepatic triglyceride levels. *P < 0.05. (**f**) Representative images of F4/80 immunofluorescence staining of mouse liver cryosections. Polyclonal antibodies against F4/80 (Green) merged with DAPI (blue). (**g**) mRNA expression of different genes in mouse livers as indicated (n = 3 for each group). (**h**) Q-RT-PCR analysis of mRNA expression of genes involved in lipogenic and fatty acid oxidation in mouse livers (n = 8). *P < 0.05, **P < 0.01.

To further study if CCDC3 plays a potential role in the regulation of metabolism under a stress condition, we fed those mice with a high-fat diet (HFD). On HFD, there were not any significant differences in their body weight, fat composition, blood glucose, and fast insulin between control and CCDC3 TG mice (Supplementary Fig. 6d–h), and neither in their blood pressure, heart rate (Supplementary Fig. 6i and j). However, their glucose intolerance and insulin resistance induced by HFD were significantly reduced in CCDC3 TG mice compared to control mice (Fig. 6a,b). Interestingly, HFD-induced fatty liver (steatosis) was also markedly alleviated in CCDC3 TG mice compared with control mice (Fig. 6c–e). As shown in Fig. 6c, after 4-month-feeding of HFD, the liver organs of the control mice were significantly bigger and paler than those of the CCDC3 TG mice, an obvious fatty liver-like appearance. Consistently, oil red O (ORO) staining also revealed that HFD-induced lipid droplets in hepatocytes are significantly decreased in the CCDC3 TG group compared to that in control mice (Fig. 6d). We also checked the liver tissues of 1-year-old mice on a standard chow diet, neither WT nor CCDC3 TG mice had developed apparent fatty liver (Fig. 6d, bottom panels on chow diet). Additionally, triglyceride content analyses showed that HFD-induced hepatic lipid accumulation is significantly reduced in CCDC3 TG mice as well (Fig. 6e). Hepatic steatosis is often associated with inflammation-related macrophage infiltration^25^. We also observed this phenomenon in the liver tissues of HFD-fed mice (Fig. 6f, upper panels on HFD) in comparison with the liver tissues of 1-yr old mice on chow diet (Fig. 6f, bottom panels on chow diet), as assessed by IHC with antibodies against F4/80^+^, a marker for macrophage infiltration. Again, interestingly, the HFD-induced macrophage infiltration was drastically reduced in CCDC3 TG mice (Fig. 6f, upper panels on HFD). These results indicate that overexpression of CCDC3 in mice can alleviate HFD-caused glucose intolerance, insulin resistance, and steatosis.

It has been shown that HFD-caused steatosis is highly associated with induction of hepatic PPAR-gamma^26–28^. Also, conditional knockout of PPAR-gamma in mouse liver demonstrated that this gene plays a prosteatostic role in the development of NAFLD^29^. Another gene called CIDEA was recently shown to play a critical role in promoting hepatic lipid accumulation and in the development of hepatic steatosis by acting as a sensor that responds to HFD^30, 31^. Thus, we determined if CCDC3 might reduce HFD-induced steatosis by influencing the expression of these genes. Interestingly, the mRNA levels of both PPAR-gamma and CIDEA were markedly decreased in CCDC3 TG mouse livers (Fig. 6g), which was in line with our observations that the HFD-induced fatty liver in CCDC3 TG mice is less severe than that in control mice (Fig. 6c–e). Also, the expression of lipogenic genes, including stearoyl-CoA desaturase-1 (SCD1), CD36, fatty acid synthase (FAS), sterol regulatory element-binding protein 1C (Serbp1C), was significantly decreased in the liver tissues of CCDC3 TG mice compared to that in control mice (Fig. 6h). By contrast, the expression of the genes involved in cholesterol metabolism (Srebp2), lipid storage (PED3B, perilipin), lipolysis (HSL) and thermogenesis (PPAR-alpha) was not altered (Fig. 6h). These results indicate that CCDC3 might prevent fat accumulation in livers by controlling the expression of transcriptional factors that are crucial for de novo lipogenesis, though the underlying mechanism remains to be studied.

### Adenoviral overexpression of CCDC3 alleviates steatosis

**Figure 7:**
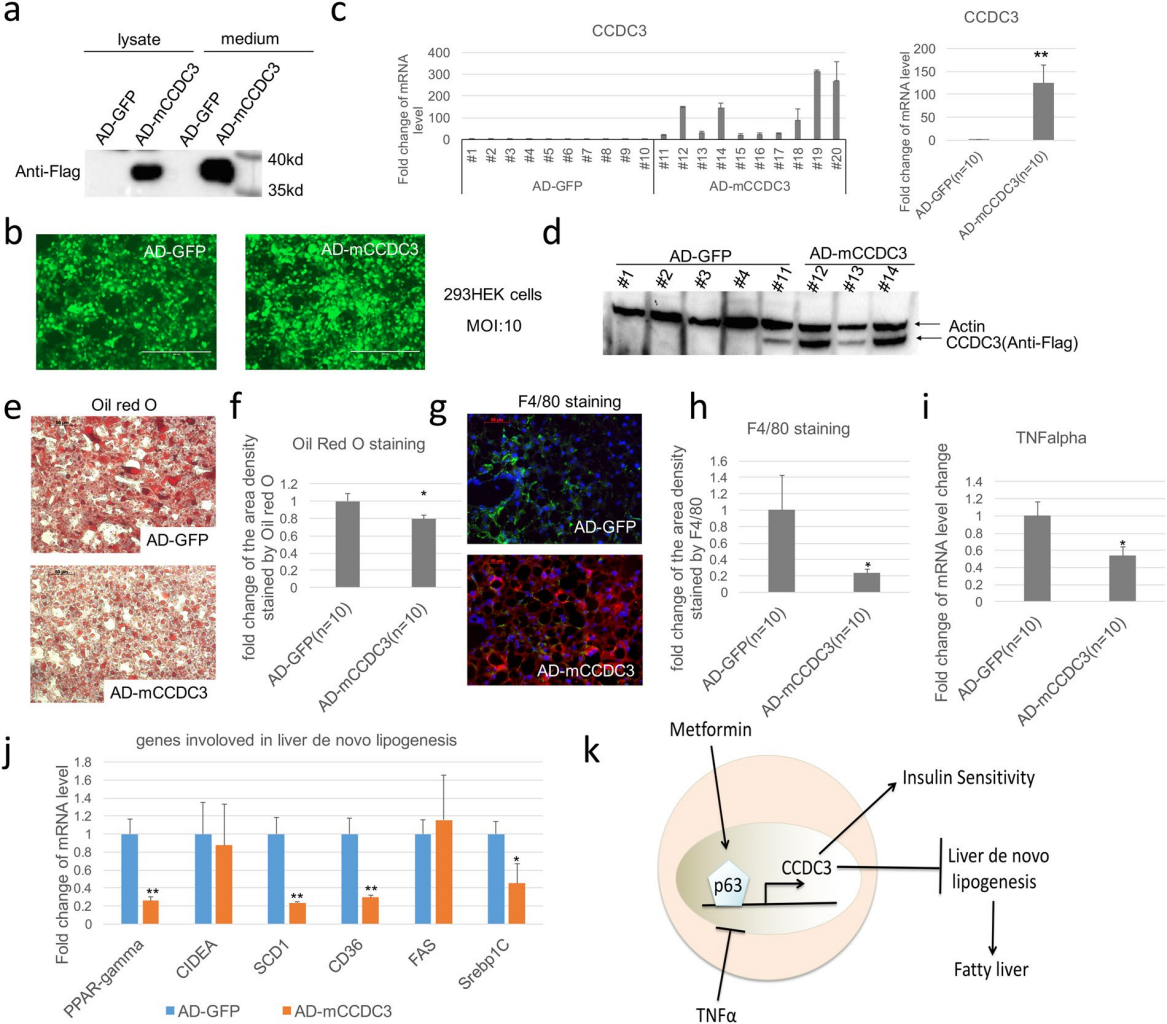
Adenovirus-mediated overexpression of CCDC3 in mouse liver reduces steatosis. (**a**) CCDC3 protein expression was confirmed in both cell lysate and medium of 293HEK cells by WB analysis. (**b**) Both of the CCDC3 expressing and control vectors express GFP after adenovirus infection (**c,d**) Real-time PCR (**c**) and WB (d) analysis of CCDC3 in mouse livers after injection with the CCDC3- or GFP-expressing virus. (**e**) Oil red O staining performed on liver sections from mice as indicated. (**f)** Quantification of the density of oil red O-stained area from panel E. (**g**) Double-immunofluorescence staining for Flag (red) and F4/80 (green) merged with DAPI (blue) of liver sections from mice as indicated. (**h)** Quantification of the density of F4/80 stained area as shown in panel G. (**i,j**) Real-time PCR analysis of TNFα (**i**) and genes involved in de novo lipogenesis (**j**) in livers of mice injected with CCDC3-expressing virus vs GFP-expressing virus. Data in panels c, f, i, and j are presented in the mean ± SD. *P < 0.05; **P < 0.01. (**k**) Schematic diagram of how the TAp63-CCDC3 pathway regulates hepatic steatosis and insulin sensitivity.

The results above (Fig. 6) suggest that CCDC3 might have a therapeutic effect on the development of fatty liver. To test this idea, we introduced adenoviruses that encode mouse CCDC3-Flag (C-terminal tag with Flag) into mice that have been fed with HFD for 3 months, by tail i.v injection as described in the Methods (Supplementary Fig. 7a). CCDC3-Flag protein expression using this adenovirus vector was confirmed in both cell lysate and medium of 293HEK cells (Fig. 7a). We chose male C57BL/6 mice for this experiment because they are highly prone to diet-induced obesity and diabetes^32^. As shown in Figs 7c and d, expression of CCDC3-Flag at both mRNA and protein levels in mouse liver tissues was confirmed by real-time PCR and WB analysis. Although the ectopic CCDC3 expression at both RNA and protein levels was remarkably high in mouse liver tissues that received adenovirus-CCDC3-Flag, compared to those that received adenovirus-GFP (Fig. 7c and d), the mice did not display any apparent behavioral adverse reaction and body weight changes after 4 weeks of virus injection (Supplementary Fig. 7b). No significant difference was found in random blood glucose (Supplementary Fig. 7c), liver weight and fat compositions (Supplementary Fig. 7d). We did not detect any change in glucose tolerance, insulin resistance between the CCDC3 vs. GFP groups of animals (Supplementary Figs 7e–h). However, ORO staining of liver tissues revealed the decrease of lipid droplets in mice expressing CCDC3 (Fig. 7e and f), which is consistent with the results from CCDC3 TG mice (Fig. 6d). Also, the CCDC3-, but not GFP-, expressing liver tissues showed a decrease of infiltrating macrophages (F4/80-positive cells) (Fig. 7g and h). Consistent with these results, the mRNA level of TNFα was significantly lower in the CCDC3-livers than that in the GFP-livers (Fig. 7i). Correspondingly, several genes involved in de novo lipogenesis, such as PPAR-gamma, SCD1, CD36, Serbp1C, except CIDEA and FAS, were significantly decreased in the CCDC3-expressing mouse livers, compared to that in GFP-expressing livers (Fig. 7j). These results indicate that CCDC3 can alleviate HFD-induced hepatic steatosis by inhibiting the expression of PPAR-gamma, SCD1, CD36, Serbp1C in a short-term period post CCDC3 injection via i.v, suggesting a therapeutic potential of this secreted protein for fatty livers, though its underlying mechanism(s) still remains to be elucidated.

## Discussion

TAp63 has been shown to play an important role in the regulation of metabolism^5, 33, 34^, as TAp63 KO mice developed obesity, insulin resistance and glucose intolerance^5^. Although Sirt1, AMPK, and LKB1 are identified as TAp63′s targets crucial for metabolism regulation^5^, it remains unknown if there are other molecules that might also facilitate the functions of TAp63 in lipid metabolism. Here, we identify CCDC3 as a novel TAp63 target gene involved in lipid metabolism. Unlike other known TAp63 targets, CCDC3 is a secreted protein that can circulate in the bloodstream and potentially target distant cells and tissues. Indeed, our further studies unveil hepatocytes as CCDC3′s target cells. Hence, this is the first time to report that CCDC3 as one of the TAp63 targets can function as an endocrine messenger involving lipid metabolism.

First, we demonstrate that CCDC3 is a bona fide target gene specific for TAp63, but not for other members of the p53 family, in both normal and cancer cells. Supporting this conclusion are the following lines of evidence. For instance, only TAp63, but no other isoforms of p63, p73 or p53, induced the expression of CCDC3 at both mRNA and protein levels (Figs 1a–c; 2a–e). Also, TAp63 bound directly to the CCDC3 enhancer region that contains p63RE DNA sequence as confirmed by both luciferase (Fig. 1d,e,f and Supplementary Fig. 1e,f) and ChIP analyses (Figs 1g and 3f). Conversely, CCDC3 level was remarkably lower in TAp63KO MEF cells and brown adipocytes than that in corresponding wild-type cells (Fig. 3d,f,g). Furthermore, ectopic expression of TAp63γ, but not p40, induced the CCDC3 expression in TAp63KO MEF cells (Fig. 3e). Moreover, p63 could regulate CCDC3 expression under physiological or stress conditions, such as the reduction of p63 transcriptional activity and the expression of CCDC3 by TNFα (Fig. 3a,b), but induction of TAp63 and CCDC3 by metformin (Fig. 3c). Lastly, the expression of CCDC3 was positively correlated with TAp63, but inversely correlated with ∆Np63, during the adipocyte differentiation (Fig. 3e), and knockdown of TAp63 in mature adipocytes led to the decrease of CCDC3 protein level (Fig. 3g and Supplementary Fig. 2c and d). Hence, these results demonstrate that the CCDC3 gene is a physiological and specific target for TAp63.

Given that CCDC3 as a secreted protein, we focused on investigating if CCDC3 might act as an endocrine molecule to target distance cells or tissues. Interestingly, we found that liver cell might serve as one main type of the target cells for CCDC3, because CCDC3 bound to the surface of liver cancer cells (Fig. 4a and Supplementary Fig. 3e). Our metabolomic and lipidomic studies using Huh7 cells also revealed that CCDC3 treatment could increase PUFAs and decrease ceramide level (Fig. 4b–g and Supplementary Fig. 4). The ability of PUFAs to prevent the development of insulin resistance has gained considerable interest in recent years^18^. PUFAs suppression of several hepatic genes involved in lipogensis was shown to be largely due to a decrease in the rate of gene transcription, such as SCD1, FAS^18^. Our studies using both cell and transgenic CCDC3 animal model systems as further discussed below showed that overexpression of CCDC3 could inhibit SCD1, FAS and other genes involved in de novo lipogenesis, which could be due to the increase of PUFAs. Ceramide is a type of lipid metabolites associated with the metabolic dysregulation that often accompanies dyslipidemia and obesity^35^. A series of studies have shown that increased ceramide level in both liver and plasma coincide with the development of liver dysfunction, hepatic insulin resistance, and steatosis in rodents^36–38^. Our results showing the increase of PUFAs and the decrease of ceramide after CCDC3 treatment suggest that CCDC3 might modify different lipid components and control insulin sensitivity. Also, we found that the decrease of ceramide synthase (CERS1) in the cells treated with CCDC3 (Fig. 4h). Those results support the idea that CCDC3 could modulate multiple lipid metabolism pathways and change the lipid profile through an endocrine action to reprogram lipid mechanism, though it remains to be determined if CCDC3 also targets other cells or tissues as suggested in Supplementary Fig. 3e, and if true, what would be the physiological outcomes.

CCDC3 TG mice displayed apparent improvement of HFD-induced hepatic steatosis compared to control mice (Fig. 6c–f). Although the underlying mechanism(s) remains to be elucidated, we found that the significant decrease of PPAR-gamma and CIDEA mRNA levels in the livers of CCDC3 TG mice compared to that in control mice must account for the this anti-steatosis phenotype (Fig. 6g). PPAR-gamma was suggested to promote insulin sensitivity and to be associated with a concomitant development of fatty liver^39^. Previous studies have shown that HFD-fed mice develop hepatic steatosis with increased PPAR-gamma expression^25, 26^. Hepatocyte-specific deletion of PPAR-gamma in mice protected HFD-fed mice from an accumulation of lipids^27, 40^. CIDEA is a PPAR-gamma target, and its deficiency alleviates the hepatic steatosis caused by HFD^30^. In line with these studies, the decrease of PPAR-gamma and CIDEA levels in the livers of CCDC3 TG mice might explain the alterations of lipid metabolism and the improvement of HFD-induced fatty livers in the animals. Our findings are also surprising because a recent study showed that knockout of CCDC3 in mice also leads to some improvement of fatty livers that are naturally developed on a standard chow diet at their 1-yr age with yet unknown mechanisms^41^. By contrast, we did not observe any apparent liver steatosis in either of CCDC3 TG or control mice on chow diet at the same age (Fig. 6d). This difference might be due to different food compositions or living environments for the mice housed in two different countries.

It is well recognized that inflammation is a physiological process highly associated with the pathogenesis of insulin resistance, type 2 diabetes, and NAFLD^42^. Macrophage infiltration in the liver has been shown to cause hepatic inflammation and steatosis^24, 43^. Interestingly, our study also suggests an anti-inflammation role for CCDC3, as we observed the marked reduction of F4/80 positive cells and the decrease of TNFα expression in CCDC3 TG liver tissues (Figs 6f; 7g,i). This anti-inflammation phenotype is in line with the previous study showing that CCDC3 decrease TNFα induced expression of vascular cell adhesion molecule-1 (VCAM-1) in endothelial cells (ECs)^11^. TNFα has been shown to contribute to the insulin resistant state^44, 45^. and genetic and pharmacological interventions that reduce TNFα functions improved obesity and insulin resistance *in vitro* and *vivo* ^46–49^. Previously, we reported that IkappaB kinase beta (IKKbeta) could suppress TAp63 activity by interfering with the interaction between TAp63 and p300^15^. Together, these studies with our present findings suggest a new TNFα-IKK-beta-TAp63-CCDC3 pathway that might offer a novel molecular insight into a better understanding of the HFD-induced hepatic inflammation and steatosis.

As mentioned above, it is surprising that CCDC3 deficiency alleviates hepatic steatosis in 1-yr old mice on a standard chow diet^41^, while the ubiquitous overexpression of exogenous CCDC3 in mice also reduces HFD-induced steatosis (Figs 5–7). In addition to the aforementioned explanations, the seemingly contradictory outcomes suggest that the underlying mechanisms for CCDC3’s physiological functions *in vivo* might be more complicated than what one could imagine. It is possible that CCDC3 might target liver cells by directly binding to its yet unidentified receptor or indirectly binding to other ligands’ receptors by forming heterodimers or hetero-oligomers with those ligands, as CCDC3 could bind to other proteins through its C-terminal coiled coil domain that has been shown to mediate protein-protein interactions^50^. If it were through the latter mechanism(s), the extremely high level of CCDC3 might negatively affect the formation of the physiological complex by sequestering its partner ligands. In this case, it might be reasonable that overexpression of CCDC3 could lead to a similar phenotype in CCDC3 knockout mice. Alternatively, because of the transgenic mice have both endogenous and exogenous CCDC3, the continuously and highly expressed ectopic CCDC3 might override the physiological function of endogenous CCDC3 by constantly occupying or sequestering the limited amounts of available CCDC3 receptors, or suppressing the expression of its endogenous receptors, hence acting as a negative regulator of the CCDC3 pathway, similar to its knockout. It is also possible that highly expressed ectopic CCDC3 might trigger other endogenous signaling pathways to suppress the physiological function of CCDC3. Our CCDC3 TG mice have normal adiposity, but also show better insulin sensitivity even when on HFD, similar to the case of TNFα knocked out mice^47^. The reason for why these CCDC3 TG animals have normal adiposity might be due to altering the expression of other cytokines when CCDC3 is overexpressed. The propensity of exogenous CCDC3 to lessen liver de novo lipogenesis would account for the reduction of fatty livers induced by HFD. All of these possibilities need to be further investigated.

Based on our studies as described here, we propose a new TAp63-CCDC3 pathway to regulate hepatic lipid accumulation. CCDC3, as a novel TAp63 target, acts as an endocrine molecule that may target hepatocytes and alleviates the pathological changes that are associated with HFD-induced steatosis, and attenuates the progression or deterioration of diseases associated with insulin resistance via an anti-lipogenesis effect in liver (Fig. 7k). Although the detailed molecular mechanisms for CCCD3 functions in hepatocytes remain largely obscure, and its receptor(s) need(s) to be identified, our studies suggest that CCDC3 without any apparently deleterious effects on mouse physiology could serve as a potential therapeutic molecule for NAFLD.

## Methods

### Cell Culture

HCT116 p53^−/−^ (RRID:CVCL_S744) cells were obtained from Dr. Bert Vogelstein at John Hopkins University School of Medicine. Human liver cancer cells HepG2 (RRID:CVCL_0027), Huh-7 (RRID:CVCL_0336), PLC-PRF-5 (RRID:CVCL_0485) were obtained from Dr. Tong Wu in Department of Pathology and Laboratory Medicine, Tulane University School of Medicine. Mouse pre-white adipocyte NIH3T3-L1 cells (RRID:CVCL_0123) and mouse pre-brown adipocyte were obtained from Dr. Sean B Lee in the Department of Pathology, Tulane University School of Medicine. Human umbilical vein endothelial cells HUVEC (RRID: CVCL_2959) were obtained from Dr. Cindy A Morris in Department of Microbiology and Immunology, Tulane University School of Medicine. Human glioblastoma SF767 cells, mouse embryonic fibroblast MEF, human osteosarcoma cells U2OS (RRID:CVCL_0042), SaoS2 (RRID:CVCL_0548), Human lung non-small-cell carcinoma H1299 (RRID: CVCL_0060) cells, human breast cancer MCF-7 (RRID:CVCL_0031), MDA-MB-231 (RRID:CVCL_0062), human embryonic fibroblast WI-38 (RRID:CVCL_0579), human embryonic kidney cell 293HEK (RRID:CVCL_0045), human cervical cancer Hela cells (RRID:CVCL_0030), human keratinocyte HaCat cells (RRID:CVCL_0038), human pancreas ductal adenocarcinoma cell Panc-1 (RRID:CVCL_0480) and Mia-paca2 (RRID:CVCL_0428) were purchased from American Type Culture Collection (ATCC). IgG-4A4 hybridoma cells (RRID:CVCL_G662) were purchased from American Type Culture Collection (ATCC).

HUVEC cells were cultured in Lonza EGM^TM^-plus growth media. MEF and WI-38 cells were cultured in Dulbecco’s modified Eagle’s medium (DMEM) supplemented with 15% fetal bovine serum (FBS), 50 U/ml penicillin and 0.1 mg/ml streptomycin (P/S). NIH3T3-L1 cells were cultured in DMEM with 10% bovine serum (calf serum) and P/S before differentiation. For differentiation, cells were seeded on day 0 and grown to confluence on poly-D-lysine coated wells for 96 hours. On day 4, the media were changed to differentiation media: DMEM high glucose with 10% FBS and P/S (basal medium) supplemented with 5 mg/L insulin, 0.25 μM dexamethasone and 0.5 mM IBMX (all from Sigma-Aldrich). After two days this medium was switched to the basal medium with only 5 mg/L insulin, and two more days later the cells were kept in the basal medium with no added hormones. All the other cells were cultured in Dulbecco’s modified Eagle’s medium (DMEM) supplemented with 10% FBS and P/S. All the cells were cultured at 37 °C in 5% CO_2_.

### Glucose and insulin tolerance tests

For the glucose tolerance tests (GTT), mice fasted for 16 h and then received intraperitoneal glucose dissolved in PBS (2 g/kg body weight). GTT was measured at 0, 30, 60 and 120 min using glucose test strips and glucose meters (Contour). For insulin tolerance tests (ITT), mice fasted for 7 h and injected intraperitoneally with insulin (Humulin, 0.75 IU/kg) purchased from Eli Lilly (Indianapolis, IN) at a final concentration of 1 U/kg body weight. Blood glucose levels were determined after that at 0, 30, 60 and 120 min.

### RNA isolation and gene expression

Supplementary Tablescted using the TRIZOL reagent (Invitrogen). For the first strand cDNA synthesis, 2 μg of total RNA was reversely transcribed at 37 °C for 1 h using oligo(dT)12–18 primers and Superscript III reverse transcriptase (Invitrogen). Real-time qPCR was performed using iTaq™ universal SYBR® Green supermix (Bio-Rad) and the CFX96 Touch™ Real-Time PCR Detection System (Bio-Rad). A standard curve was used to calculate mRNA level relative to that of the control gene, 36B4 (mouse) or GAPDH (human). All primers were synthesized by Invitrogen. Primers for the various genes are listed in Supplementary Table 1.

### Western blot analysis and Antibodies sources

*In vitro* cultured cells or isolated liver tissues were homogenized in lysis buffer containing protease/DTT/PMSF and centrifuged (12,000 *g*, 15 min, 4 °C). Supernatants were mixed with 4 × SDS-PAGE sample buffer, and the protein concentration of homogenates was determined by the Bradford method. Equal amounts of protein were subjected to SDS-PAGE and transferred to polyvinylidene difluoride (PVDF) membranes. Detection was carried out using antibodies against CCDC3 (GeneTex, GTX81055, 1:500 dilution), Beta-Actin (Santa Cruz, Sc-47778, 1:1000 dilution), Flag (Sigma, F3165-2MG, 1:2000 dilution), human p21 (Santa Cruz, Sc-471, 1:300 dilution), mouse p21 (Santa Cruz, Sc-6246, 1:200 dilution), GFP (Santa Cruz, Sc-9996, 1:1000 dilution), β-Tubulin (Santa Cruz, Sc-9104, 1:1000 dilution), IKBα (Cell Signaling, #8414, 1:1000 dilution), Sirt1 (Santa Cruz, sc-15404, 1:1000 dilution), and puma (Santa Cruz, sc-377015) respectively. p63 antibody used for Fig. 3f,g were purified from cell culture supernatant of the IgG-4A4 hybridoma cells (ATCC® CRL-1898™) using protein A affinity purification, all the other figures except Fig. 3f,g were used p63 antibody from Santa Cruz (Sc-8431, 1;1000 dilution).

### *In vitro* lentivirus infections

shTAp63 and control lentivirus vectors were obtained from Elsa R Flores. 3T3-L1 preadipocytes were cultured 2.5 days after differentiation, infected with virus-containing media supplemented with 2 μg/ml polybrene for 48 h. Cells were harvest for Western-blot analysis at Day 4.5 after differentiation (post 2days after virus infection).

### Liver triglyceride determination

Liver tissues were freshly cut at 200 mg per piece and stored at −80 °C until use. The tissues were homogenized in 1 ml 5% Triton-X100 on the ice and centrifuged at 10,000 × g for 10 minutes at 4 °C, and diluted at the 1:5 ratio in extraction buffer before use for analyses. The triglyceride concentration was determined by using a commercial Triglyceride Assay kit (#BE10A31) from EnzyChrom^TM^.

### Determination of blood chemistry

Blood samples were collected and centrifuged at 2,000 G for 20 min to isolate sera. Blood triglyceride was measured using a commercial Triglyceride Assay kit (#BE10A31) from EnzyChrom^TM^. Insulin levels were determined using an Ultra-Sensitive mouse insulin ELISA kit (#12MAUM215) purchased from Crystal Chem. INC.

### Analysis of hepatic lipid content by Oil Red O staining

The Oil Red O(ORO) stock solution consisted of 0.5% ORO in isopropyl alcohol. OCT-embedded liver samples were cut into 7 μm sections and stained with freshly diluted ORO(4 volumes of water to 6 volumes of ORO stock solution) to evaluate the hepatic lipid content. Briefly, cryosections were fixed in 60% isopropanol for 10 min and stained in working ORO solution for 10 min at 60 °C and subsequently washed in 60% isopropanol. Sections were counterstained with Gill’s hematoxylin for 30 sec, washed thoroughly in running tap water for 3 min, and mounted with the aqueous solution. Sections were visualized under an Axiovert 200 M microscope (Carl Zeiss) at ×20, and relative areas of steatosis were expressed as percentage ORO staining.

### Detection of F4/80 by immunohistochemistry

The cryosections of liver tissues were boiled in fresh citrate buffer (10Mm Sodium Citrate,0.05% Tween-20, pH6.0) using a steamer for 35 min for antigen retrieval. After being blocked with blocking buffer (5% Goatserum, 0.3% TritonX-100 in 1xPBS) for 1 h at RT. Sections were incubated with the primary rat-anti-rabbit F4/80 antibody (Proteintech, 18705-I-AP, 1:200 dilution), in a humid chamber at 4 °C overnight, followed by incubation for 40 min at room temperature with HRP-conjugated goat anti-rabbit IgG secondary antibody (1:200 dilution). Sections were visualized under an Axiovert 200 M microscope (Carl Zeiss) at ×20.

### Metabolic profiling and data analysis

For metabolic profiling, all samples were shipped on dry ice to the NIH West Coast Metabolomics Center, University of California in Davis. For gas chromatography time-of-flight (GC-TOF) mass spectrometry (MS) analysis, samples were randomized before analytic analysis by using the Laboratory Information Management System, MiniX. For liquid chromatography. The data were further processed with normalization, scaling, filtering, and statistical analysis using MetaboAnalyst 3.0 (www.metaboanalyst.ca) with T-test cut off, p < 0.05; and two-fold change cut off. The data were mean centered, square root scaled, and normalized, such that the sum of squares for each chromatogram equaled on statistical analysis. Insignificant features between samples treated with CCDC3 conditioned medium and control medium were filtered out using both univariate and multivariate analyses. For multivariate analysis, principal component analysis (PCA) was performed for unsupervised analysis, while partial-least-squares discrimination analysis (PLS-DA) was performed for supervised analysis to identify features with discriminative power. A score plot was applied to reduce the dimensionality of the data for grouping of the samples, in which each point in the score plot represented the individual sample, and similar data sets exhibited clustering, while different sets separated farther apart. PLS-DA models were validated based on accuracy, the multiple-correlation coefficient (*R* ^2^), and cross-validated *R* ^2^ (*Q* ^2^) in cross-validation. The significance of the biomarkers was ranked using the variable importance in projection (VIP) score (>1) from the PLS-DA model that was responsible for the separation of CCDC3 conditioned medium treated samples from control samples.

### Generation of CCDC3 transgenic mice

With the primers containing the coding sequence for Ccdc3 signal peptide and 3X Flag tag, mouse Ccdc3 was amplified by PCR from pCDNA-his/myc-Ccdc3 plasmid, digested with EcoRI and ligated into pMES-STOP expression vector. The recombination plasmid with correct orientation was confirmed by DNA sequencing and designated as PMES-mCCDC3 (data not shown). Ccdc3 transgenic mice were generated by microinjection of DNA fragment from PMES-mCcdc3 into the pronuclei of fertilized single-cell mouse embryos using standard techniques at the Tulane Transgenic Animal Facility. Hybrid mouse strain B6D2F1/Hsd, supplied by the facility, was used as the source of embryos for the micro-manipulation. Three Ccdc3 conditional overexpression founders were identified by PCR-based genotyping performed on tail-extracted genomic DNA. Transgenic mice with global overexpression of mouse Flag-CCDC3 were generated by crossing the founders with CMV-Cre mice (C57BL/6) to remove the transcription stop cassette. The CCDC3 transgenic mice were mated to C57BL/6 mice for 3 more generations before characterization.

All animal experiments were conducted in accordance with the National Institutes Health “Guide for the Care and Use of Laboratory Animals” and approved by the Institutional Animal Care and Use Committee at Tulane University School of Medicine (protocol number:4257R). Male CCDC3 TG/WT mice were used throughout this study and maintained (5 mice/cage) on a 12 h light/12 h dark daily cycle and fed a standard chow diet or high-fat Diet (HFD) (RD western diet # D12079B, Research Diets).

### Genotype analysis by PCR

PCR was performed using tail genomic DNA to further determine if CCDC3 is integrated into the mouse genome. The sequences of primers used to amplify a 503-bp fragment of the CCDC3 transgene were listed in Supplementary Table 2. PCR conditions were as follows: pre-denaturation at 94 °C for 10 min, followed by 30 amplification cycles of denaturation at 94 °C for 1 min, primer annealing at 58 °C for 1 min, and extension at 72 °C for 30 s, and finally an additional extension at 72 °C for 10 min.

### CCDC3 expression using adenoviral delivery

The full-length mouse CCDC3 cDNA containing FLAG-tag encoding sequence was subcloned into p-shuttle-1-IRES-GFP vector. The CCDC3 plasmid was then recombined into pAd -easy-1 virus vector. CCDC3 and GFP recombinant adenoviruses (Ad-CCDC3 and Ad-GFP, respectively) were produced in HEK293T cells and purified by cesium chloride gradients centrifugation. C57/B6 WT mice were infected with Ad-CCDC3 or Ad-GFP (control) with doses of 10^9^ pfu/mice via tail vein injection. Mice at 28 days post-infection were analyzed.

### Statistics

The data were expressed as the mean ± SD. Statistical analysis was performed using the Student’s t-test, and a *P* value below 0.05 (*P* < 0.05) was considered significantly different.

## Electronic supplementary material

Supplementary Information
