# Supplementary material for: Ccdc3: A New P63 Target Involved in Regulation Of Liver Lipid Metabolism
Source: Sci Rep. 2017 Aug 21;7:9020. doi: 10.1038/s41598-017-09228-8 (PMC5566403; doi:10.1038/s41598-017-09228-8)
Supplement: Supplementary file 1 — Supplementary Information [file 41598_2017_9228_MOESM1_ESM.pdf]

Supplementary Information

CCDC3: A NEW p63 TARGET INVOLVED IN REGULATION OF LIVER LIPID METABOLISM

Wenjuan Liao[1], Hongbing Liu[1,2#], Yiwei Zhang[1#], Ji Hoon Jung[1], Jiaxiang Chen[1,3], Xiaohua Su[4], Yeong C. Kim[5], Elsa R Flores[4], San-ming Wang[5], Malwina Czarny-Ratajczak[6], Wen Li[7], Shelya X. Zeng[1*] and Hua Lu[1*]

[1]Department of Biochemistry & Molecular Biology and Tulane Cancer Center, [2]Department of Pediatrics (current address), and [6]Department of Medicine, Center for Aging, Tulane University School of Medicine, New Orleans, LA, 70112, USA; [3]Department of Physiology, Jiangxi Medical College of Nanchang University, Nanchang, Jiangxi 330006, P.R. China (current address); [4]Department of Molecular Oncology, Cancer Biology and Evolution Program, H. Lee Moffitt Cancer Center, Tampa, Florida, USA; [5]Department of Genetics, Cell Biology and Anatomy, University of Nebraska Medical Center, 986805 Nebraska Medical Center, Omaha, NE 68198, USA; [7]Laboratory of General Surgery, The First Affiliated Hospital, Sun Yat-sen University, Guangzhou, Guangdong 510080, P.R. China.

Running title: Regulation of liver lipid metabolism by CCDC3.

#: Equal contributions.

*Co-correspondence:

Hua Lu, Department of Biochemistry and Molecular Biology, Tulane Cancer Center, Tulane University School of Medicine, 1430 Tulane Ave., New Orleans, Louisiana, 70112, USA. Email: hlu2@tulane.edu.

Shelya X Zeng, Department of Biochemistry and Molecular Biology, Tulane Cancer Center, Tulane University School of Medicine, 1430 Tulane Ave., New Orleans, Louisiana, 70112, USA. Email: szeng@tulane.edu.

	Forward primer	Reverse primers
h-CCDC3	ACAGTAGGCTCATGTGCTCCTCGG	TGCCGGTTGCGCTTCTCCAG
h-GAPDH	GATTCCACCCATGGCAAATTC	AGCATCGCCCCACTTGATT
h-CIDEA	TTCAGACCTTGGGAGACAAC	TCAGCCTGTACAAGTCGAAG
h-SREBF1C	GCAGATCGCGGAGCCATGGATTGC	GAGGTGGAGACAAGCTGCCTGG
h-CERS1	ATCGTCTCCTCCTACGCCTT	AACCAGAACCAGCTGAAGCC
h-HMGCS1	TGTCCTTTCGTGGCTCACTC	TGAAAGAGCTGTGTGAAGGAT
m-CCDC3	TGGTCCAGGACTACTCTTATTTCTT	GAAAACATCCTTCTGTTCTCCTG
m-PPAR-gamma	TTGTGAAGGATGCAAGGGTTT	CTTCTCCTTCTCGGCCTGTG
m-CIDEA	TGCTCTTCTGTATCGCCCAGT	GCCGTGTTAAGGAATCTGCTG
m-SCD1	CTGTACGGGATCATACTGGTTCCC	CAGCCGAGCCTTGTAAGTTCTGTG
m-CD36	TCGGATCTGAAATCGACCTT	CACAGGCTTTCCTTCTTTGC
m-FAS	CTGAGATCCCAGCACTTCTTGA	GCCTCCGAAGCCAAATGAG
m-SREBP1C	GGAGCCATGGATTGCACATT	GGCCCGGGAAGTCACTGT
m-SREBP2	GATGAGCTGACTCTCGGGGACATC	GTGGGGTAGGAGAGACTTTGACCT
m-PDE3B	ATGAGGAAAGACGAGCGCGA	CGGCAGAGGTGGAAGAAGAA
m-Perilipin	AGAGTTCTGCAGCTGCCTGTG	CAGAGGTGCTTGCAATGGGCA
m-PPAR-alpha	TCTCCACGTTCCAGCCCTTCCTCA	TTCACATGCGTGAACTCCGTAGTG
m-36B4	GCTCCAAGCAGATGCAGCA	CCGGATGTGAGGCAGCAG
mTAp63	AACCCCAGCTCATTTCTC	GGCCCGGGTAATCTGTGTTGG

Supplementary Table 1. List of Primers for qRT-PCR and semiquantitative RT-PCR

	Forward primer	Reverse primers
CCDC3 TG mice genotyping	TTCTTTTTCCTACAGCTCCTG	TAGAAGAGCCCTCCCTCTCC
CCDC3-BS1(luciferase reporter assay)	TGAGACCATTGCCTCTGCTTCTGAG	CACCCTGCCAGAAGGTGCCTG
CCDC3-BS2(Luciferase reporter assay)	CCCCCAGCTGGTGCATCGAC	GTCAACACAAGCCAAGGATTGAGGA
CCDC3-BS1 （ChIP）	AGGGCGTCTCTGTGAAGGGCA	GCCACACTTTTAAACCGTGTCTCCC
CCDC3-BS2 （ChIP）	TGGCCAACATGGCAAAAA	ACTACAAGCGCATGCCATCA
CCDC2-3'UTR （ChIP）	CCCCTCCAGGGGACAGATCCCA	GGCTCTTTGCCAGTTGCCTGGTT
CCDC3-mBS1(ChIP)	TTACTTTCTCCCTCCATTTTCTCC	CAGGAAGAAGATTGTCATAGGGA
CCDC3-mBS2(ChIP)	GGCTTTGTTGGTGCTCTGAC	AGTGTTTGGTTTTGTTTGTTTGAA
CCDC3-m-3'UTR(ChIP)	ATGCCTCATCCTCCTGGTCT	CAGGGGCAGAGCTTAGTTGT
mp21(ChIP)	GACCCCAAAATGACAAAGTGACAA	CCTTTCTATCAGCCCCAGAGGATACC

Supplementary Table 2. List of Primers for CCDC3 TG mice genotyping, luciferase reporter assay and chromatin immunoprecipitation

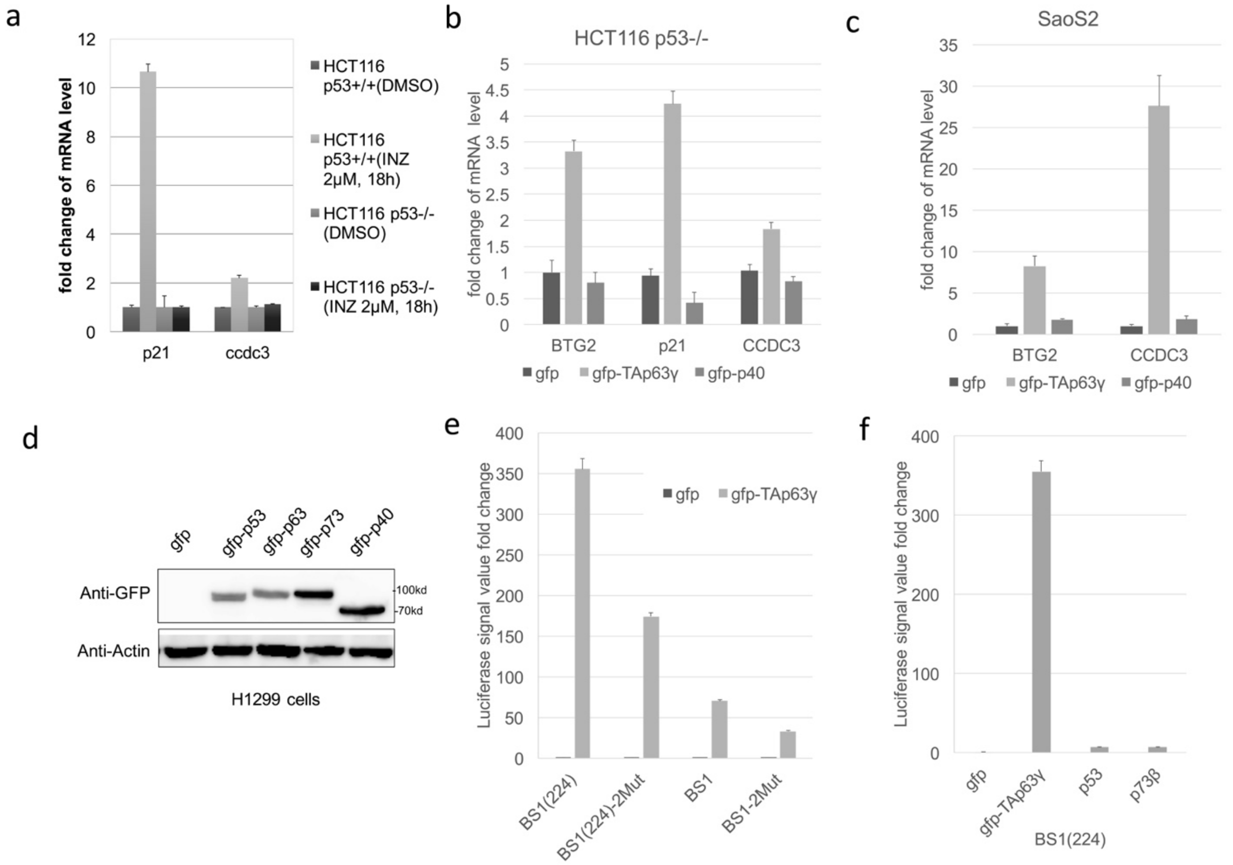

Supplementary Fig. 1: (**a**) INZ induces the expression of mRNA of CCDC3 as well as p21 in $p53^{+/+}$ HCT116 cells, but not $p53^{-/-}$ HCT116 cells. (**b, c**) qRT-PCR showing that overexpression of TAp63 could induce CCDC3 as well as p21 and BTG2 in $p53^{-/-}$ HCT116 cells (b) and SaoS2 cells (c). (**d**) WB analysis showing that ectopic protein expression levels of p53, p63, p73 and p40 in H1299 cells in the luciferase assay. (**e, f**) H1299 cells were co-transfected with the indicated plasmids and β-Gal plasmid. 48 hours after transfections, cells were harvested for luciferase assays. Luciferase activity was normalized against β-Gal expression. Data are presented as means ± S.D., n = 3

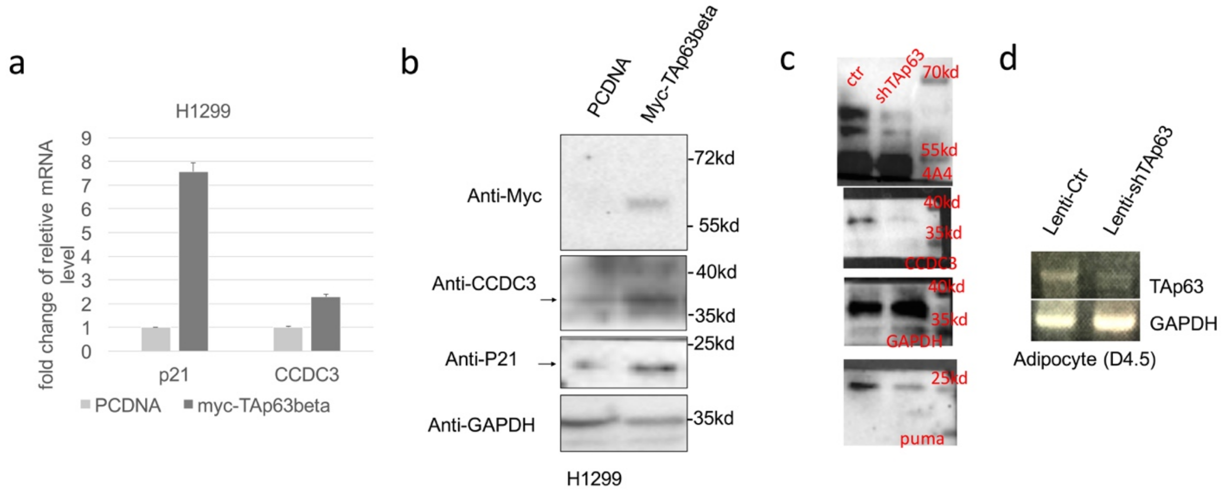

Supplementary Fig. 2: qRT-PCR(**a**) and WB analysis(**b**) showing that overexpression of TAp63beta in H1299 cells can induce CCDC3 level. (**c**) Original western blots for Fig. 3g. (**d**) Semiquantitative RT-PCR for TAp63 and GAPDH.

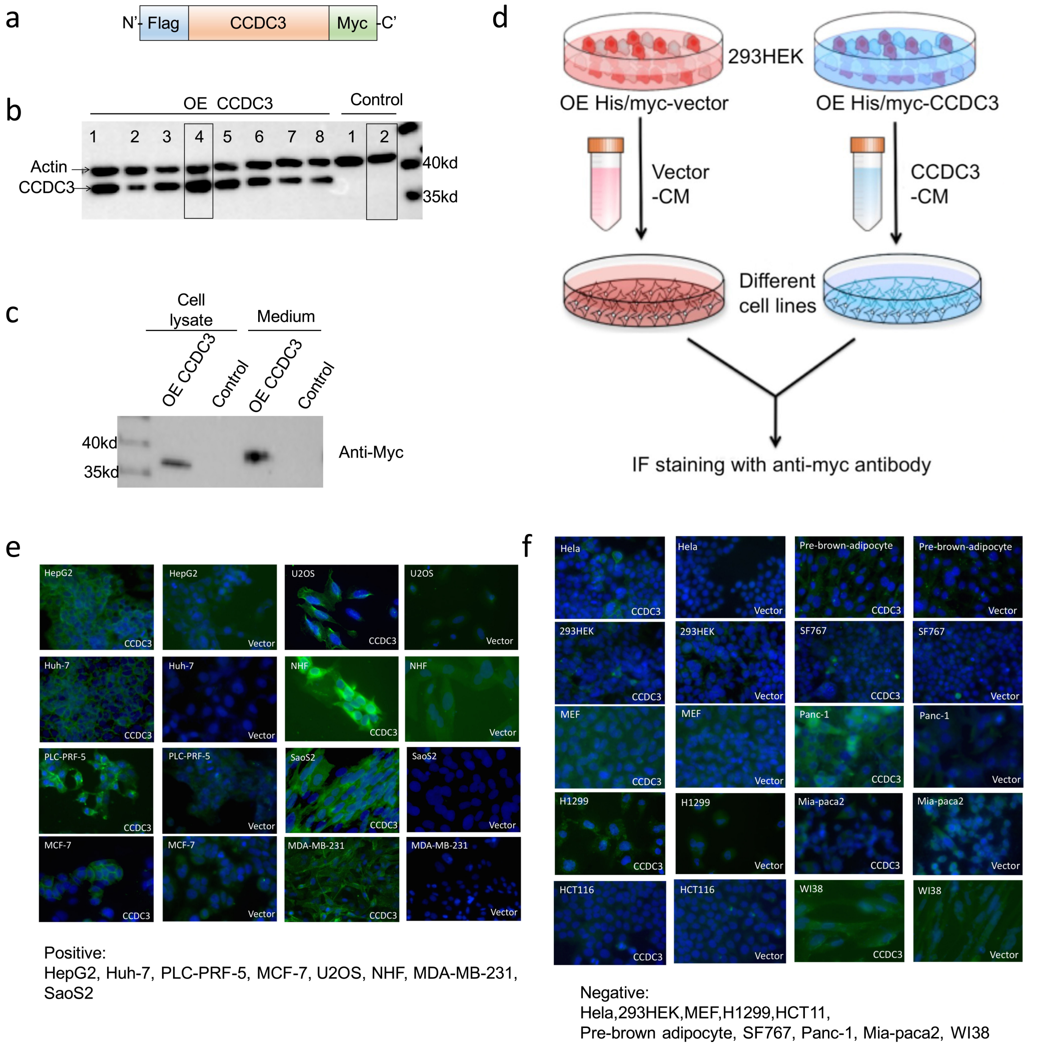

Supplementary Fig. 3: (**a**) Schematic diagram of ectopic CCDC3 with N'-terminal Flag tag and C'-terminal Myc tag. (**b**) Screening 293 stable cells with overexpress CCDC3 (OE CCDC3) or control vector, clone 4 in OE CCDC3 and clone 2 in OE control were picked and used in this study. (**c**) WB analysis of CCDC3 expression in both cell lysate and medium in 293 stable cells with OE CCDC3 or Control vector. (**d**) Schematic graph showing that different cells were cultured with CCDC3 conditioned medium (CM) and control medium, and followed by immunostaining with anti-Myc antibody as indicated. (**e**, **f**) Screening various human and mouse cells that might be recognized by CCDC3-Myc as detected by IF staining with anti-Myc antibodies. Myc-positive cells are shown in panel e, but Myc-negative cells are shown in panel f.

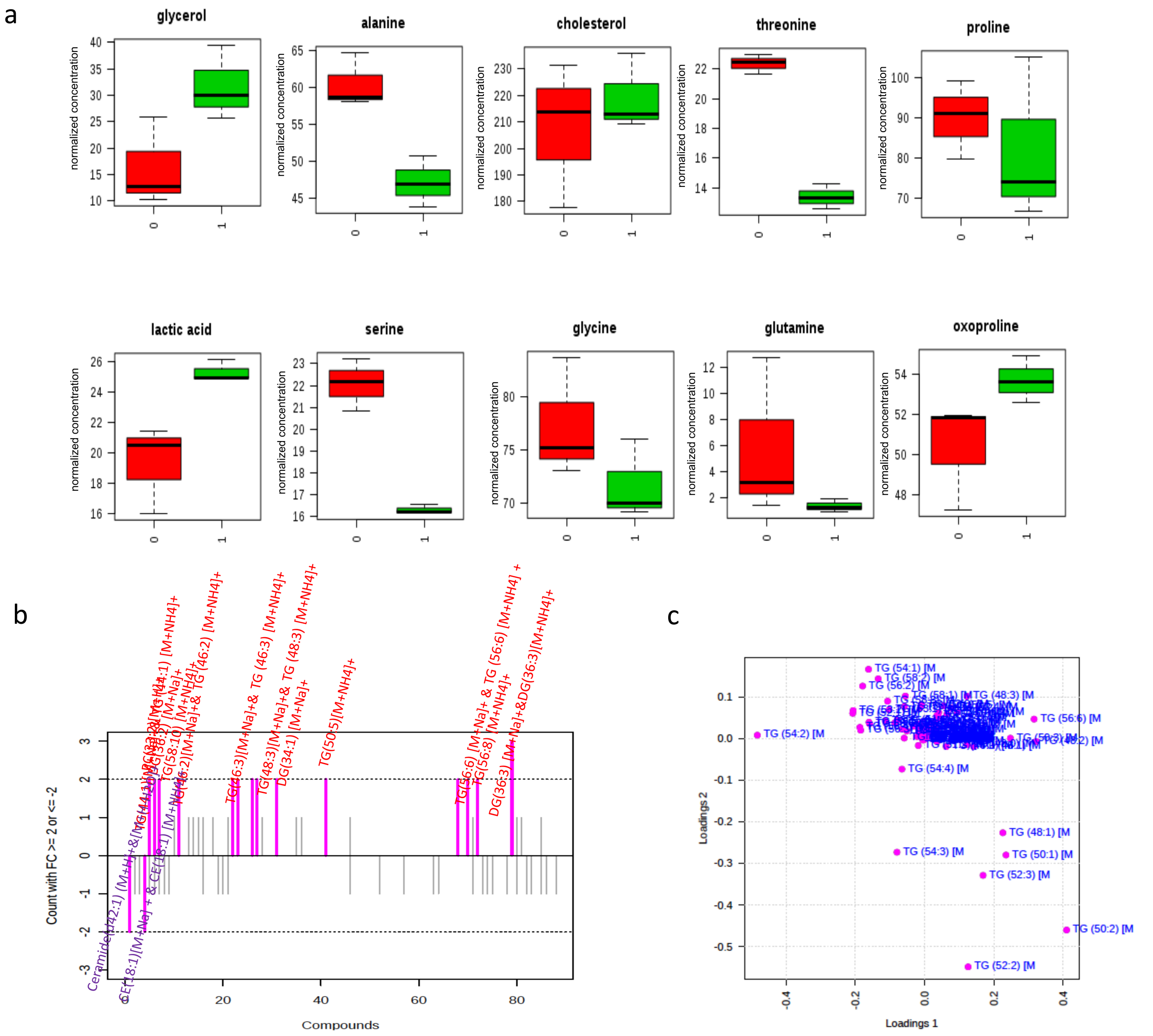

Supplementary Fig. 4: (**a**) Top 10 metabolites changed under CCDC3-CM culture. (**b**) Important features selected by fold-change analysis with threshold 1.5. The pink bars represent features above the threshold. (**c**)PLS-DA loading plot comparing lipids between vector-CM (loadings 1) and CCDC3-CM (loadings 2) treated Huh7 samples. The lipid metabolite in cell samples with Variable Importance in Projection (VIP) values of more than1.0 are marked in pink.

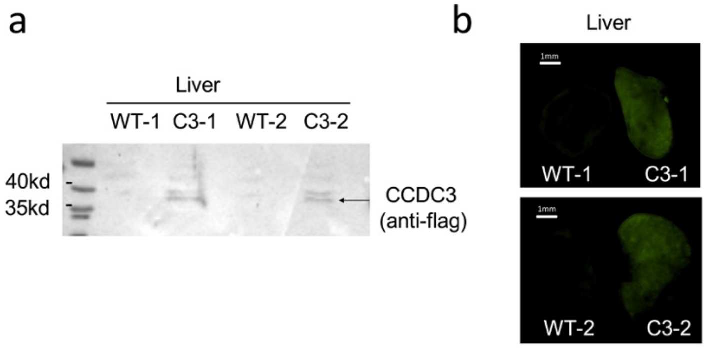

Supplementary Fig. 5: (**a)** WB analysis confirms the CCDC3 protein express in liver tissue of the two days old TG mice (**b**) Representative images of GFP expression in the liver tissue isolated from the two days old mice, taken under an anatomic microscope.

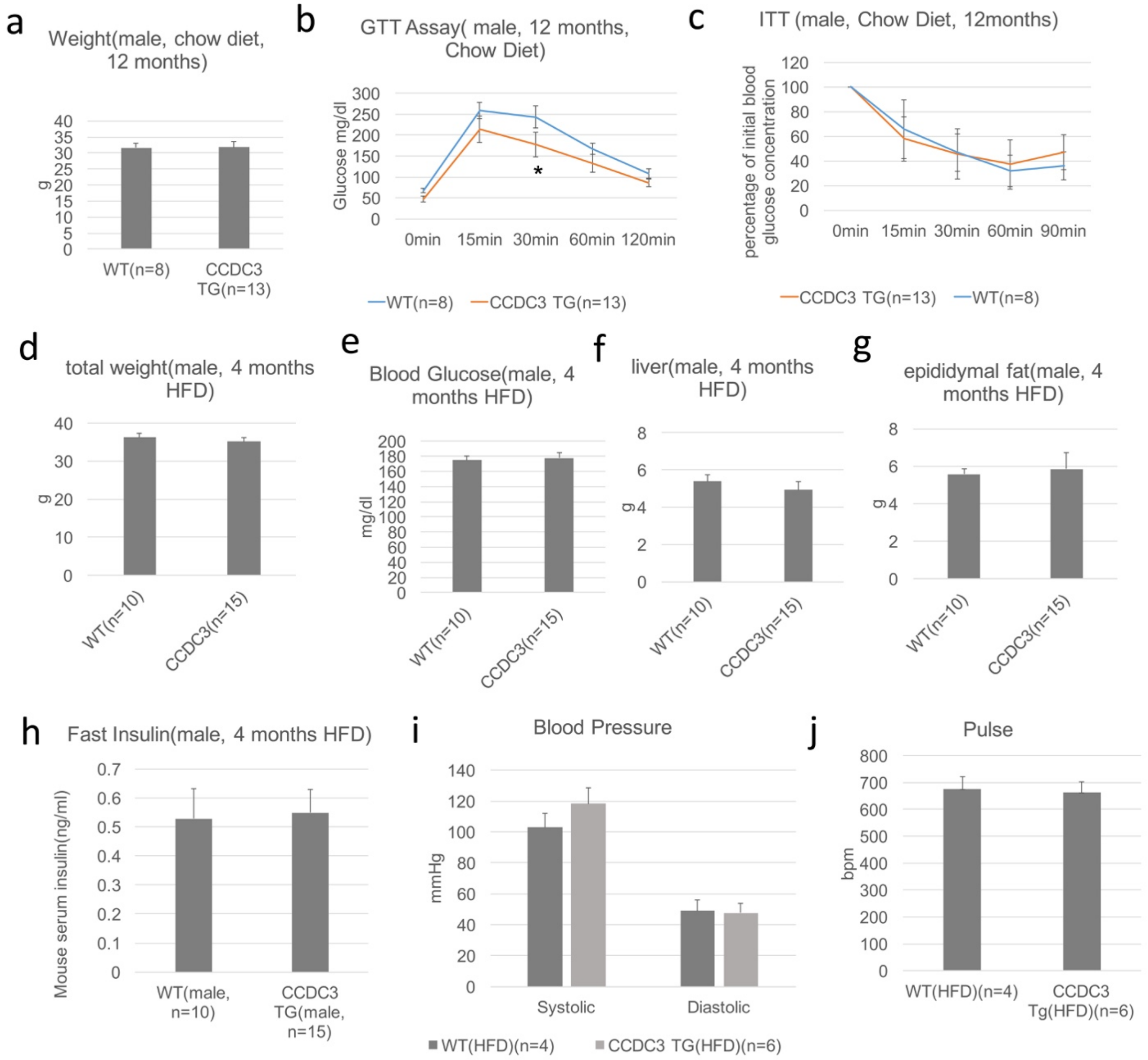

Supplementary Fig. 6: (**a**) Total body weight of both wild-type and CCDC3 TG groups of mice on chow diet. (**b, c**) Glucose tolerance test (b) and Insulin intolerance test (c) performed on wild-type and CCDC3 TG mice after 12 months on chow diet. (**d-j**) Total body weight (d), blood glucose(e), liver weight (f), epididymal fat (g), fast insulin (h), blood pressure(i) and pulse (j) of control and CCDC3 TG mice 4 months after being fed with HFD. Data represent the mean ± SD. *P<0.05.

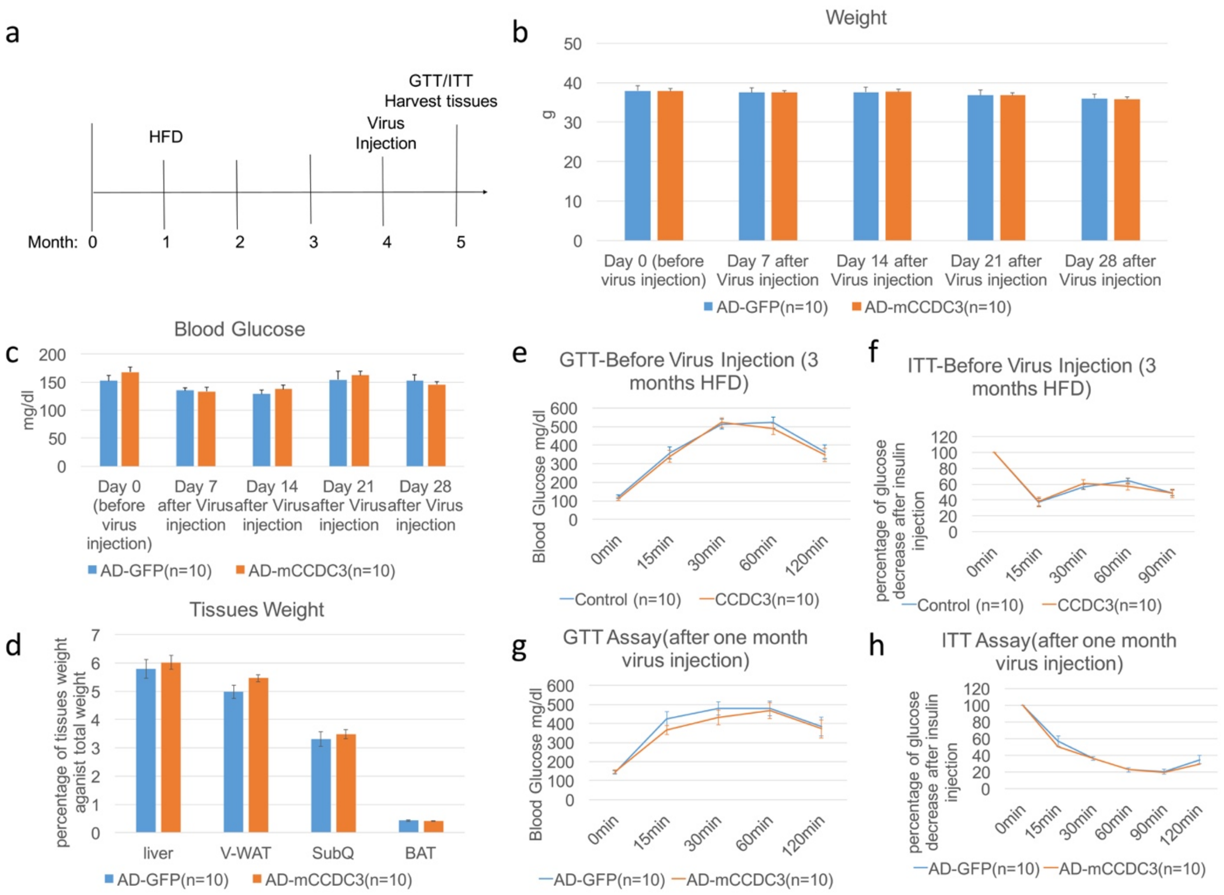

Supplementary Fig. 7: (**a**) Schematic diagram of the experimental schedules for adenovirus-CCDC3 or adenovirus-GFP injection in mice on HFD. (**b-d**) Total body weight (b), blood glucose(c) and the percentage of different tissue weight against total weight (d) at each week after mice injected with control or mCCDC3 virus. Data are presented in the mean ± SEM (n = 10 per group). (**e-h**) Glucose tolerance test (e, g) and Insulin intolerance test (f, h) performed on mice before (e, f) and after (g, h) injected with control or mCCDC3 virus. Each point on the graph indicates the level of glucose in the blood in GTT assay or the percentage of glucose decrease after insulin injection in ITT assay at the indicated time point on the x-axis.
